# Supplementary figures and images for: Analysis of intracellular communication reveals consistent gene changes associated with early-stage acne skin
Source: Cell Commun Signal. 2024 Aug 14;22:400. doi: 10.1186/s12964-024-01725-4 (PMC11325718; doi:10.1186/s12964-024-01725-4)

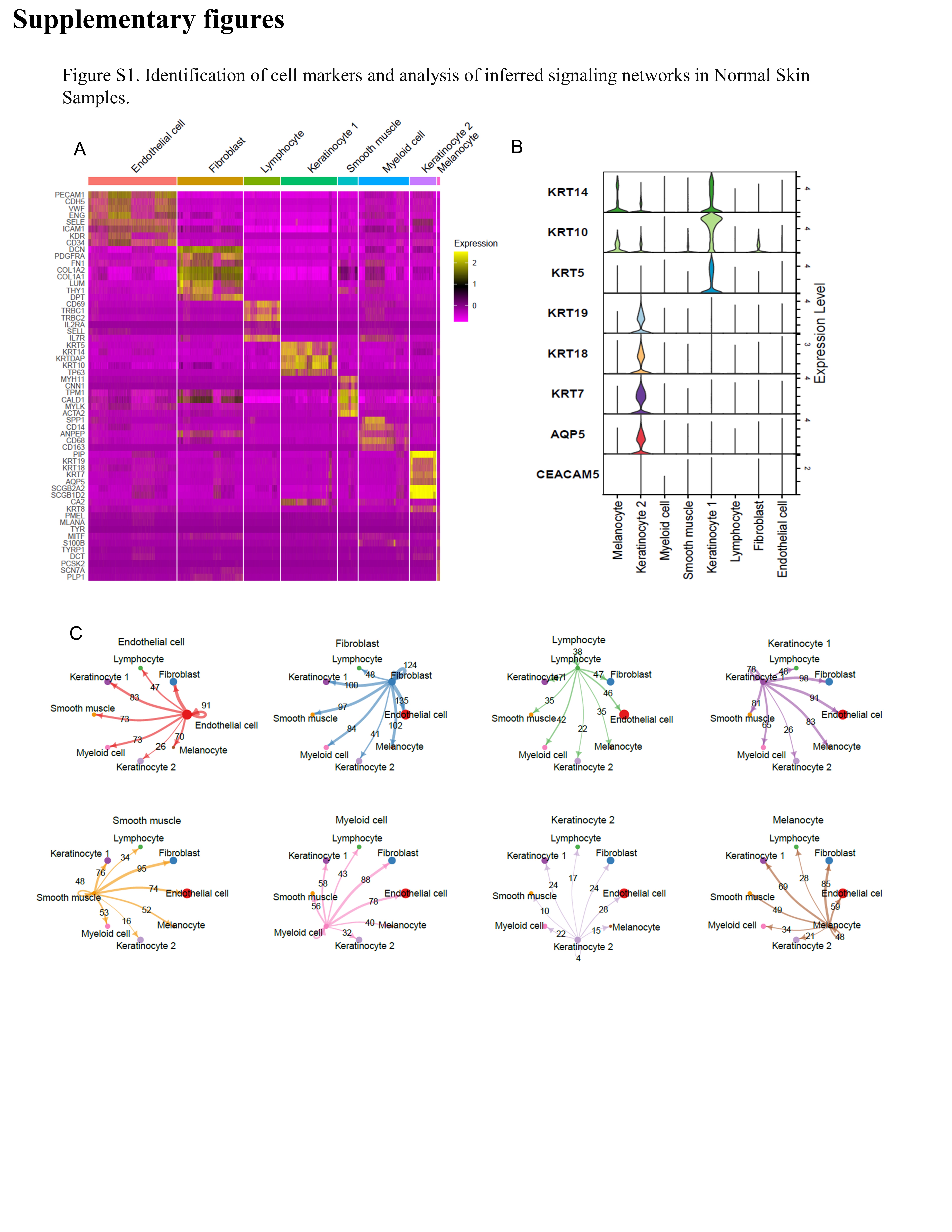

Supplement: Supplementary file 1 — Supplementary Material 1 [file 12964_2024_1725_MOESM1_ESM.jpg]

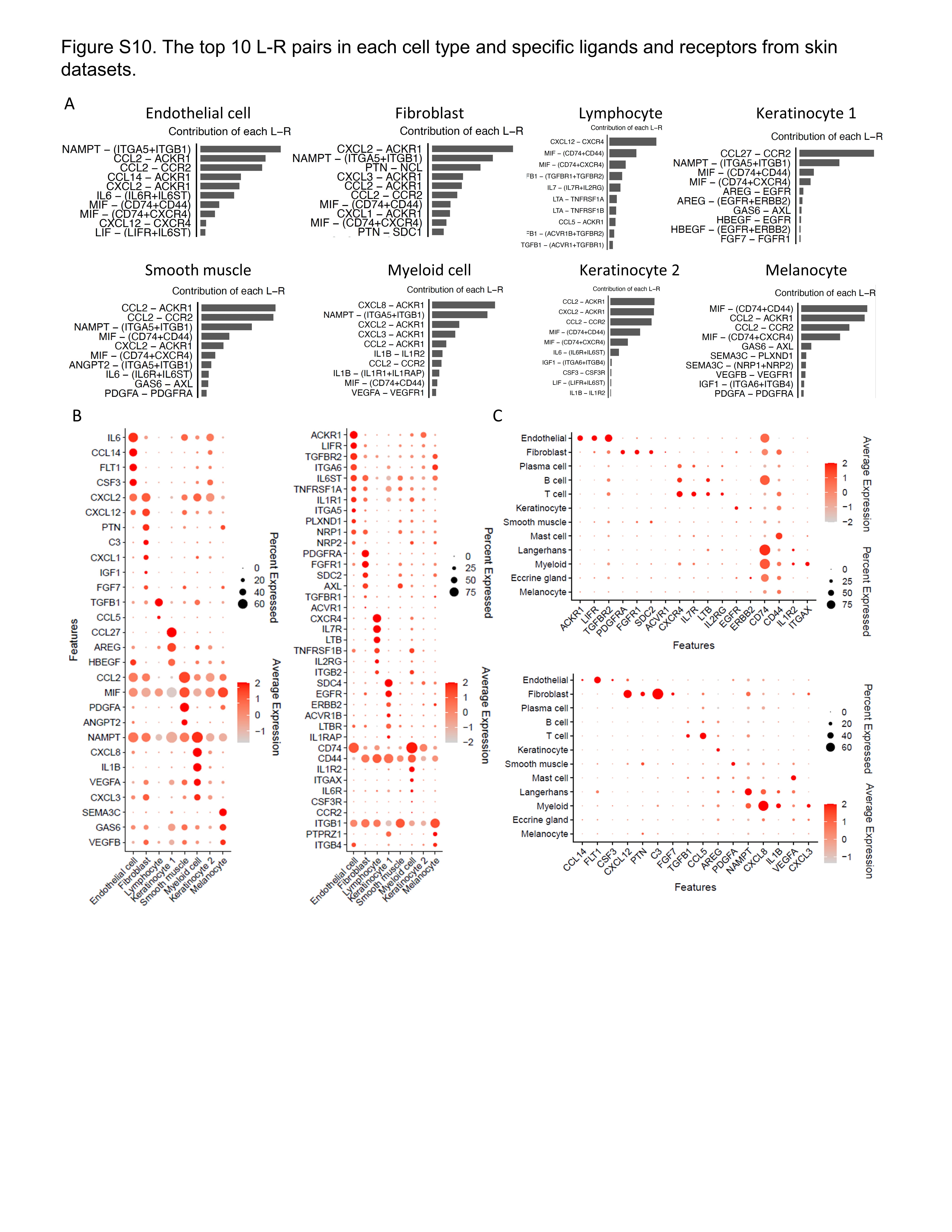

Supplement: Supplementary file 3 — Supplementary Material 3 [file 12964_2024_1725_MOESM3_ESM.jpg]

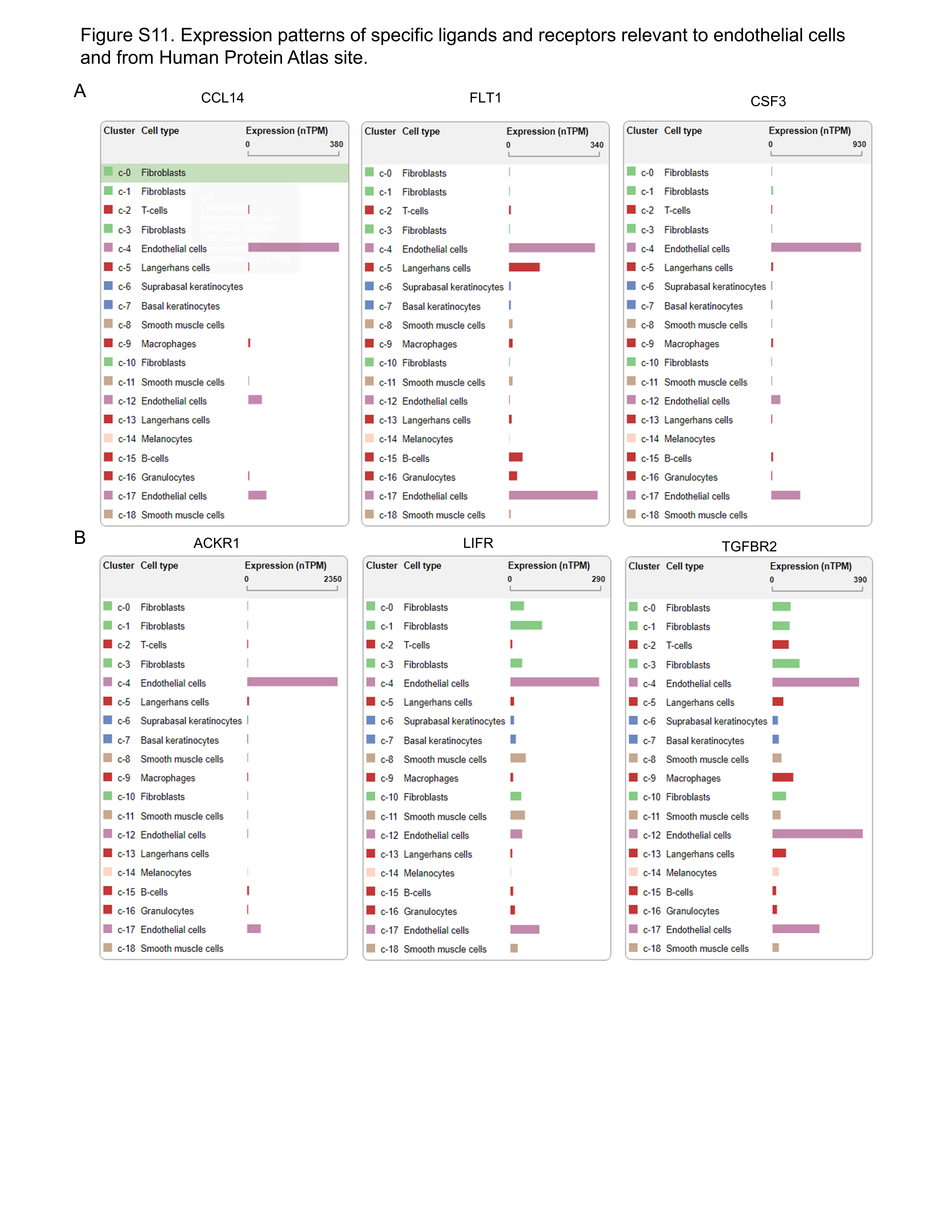

Supplement: Supplementary file 4 — Supplementary Material 4 [file 12964_2024_1725_MOESM4_ESM.jpg]

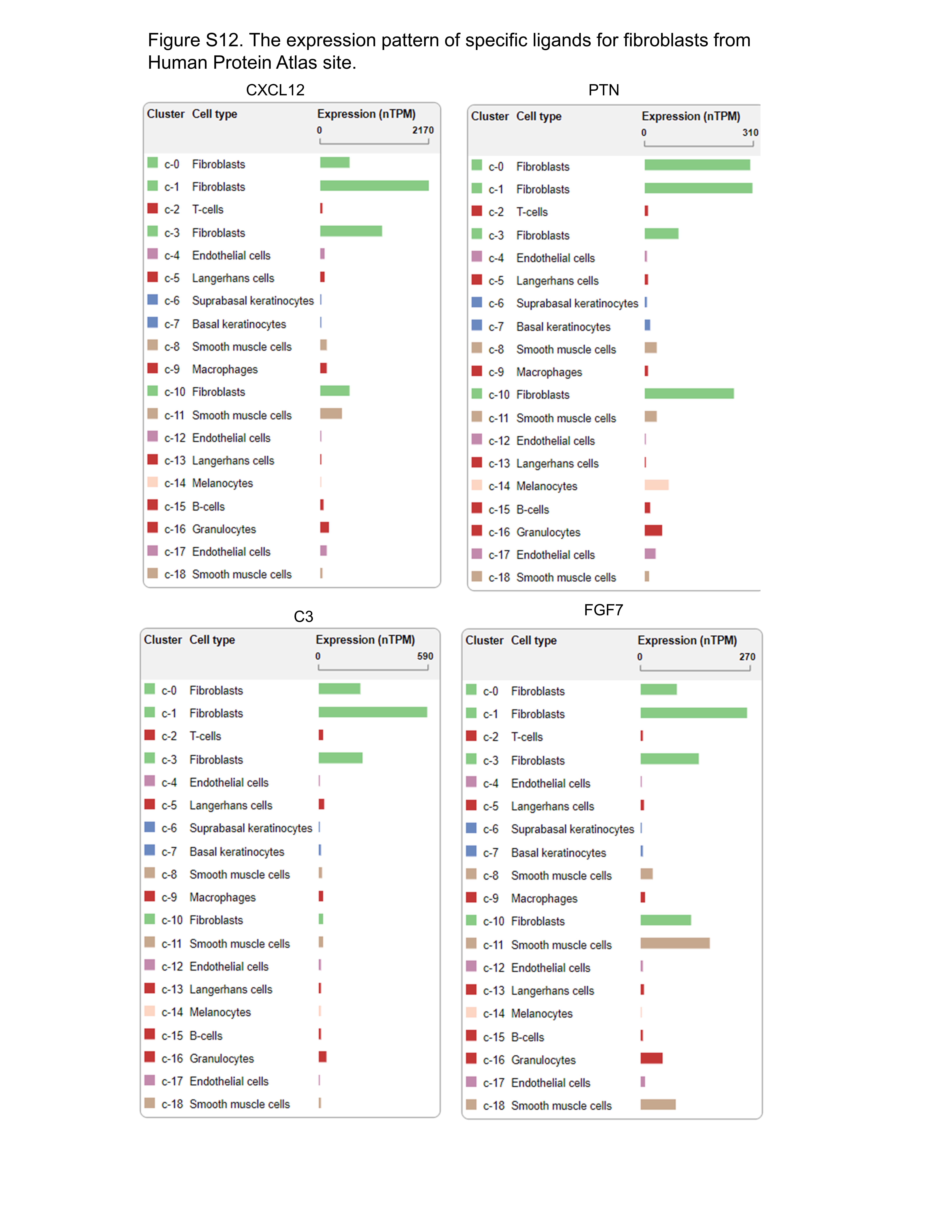

Supplement: Supplementary file 5 — Supplementary Material 5 [file 12964_2024_1725_MOESM5_ESM.jpg]

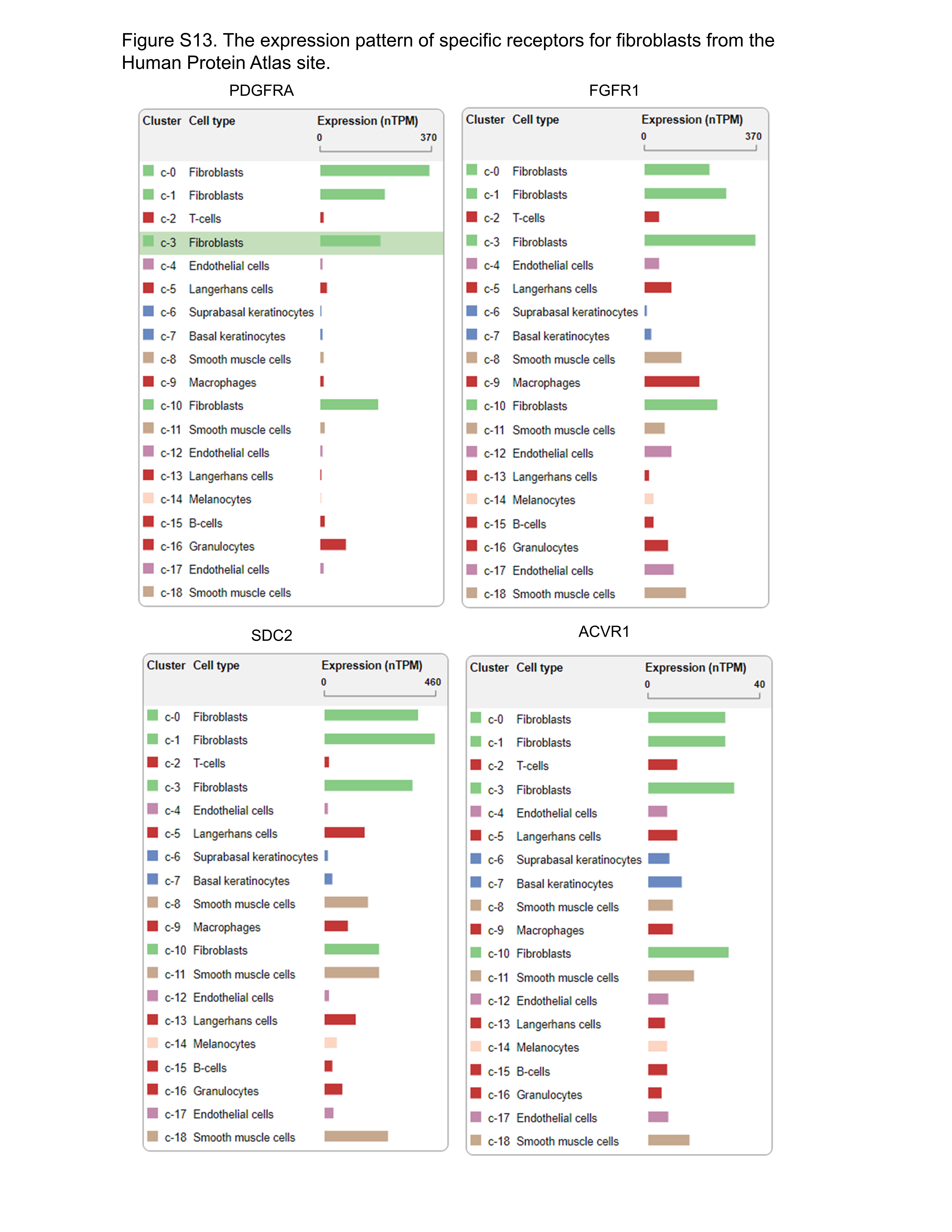

Supplement: Supplementary file 6 — Supplementary Material 6 [file 12964_2024_1725_MOESM6_ESM.jpg]

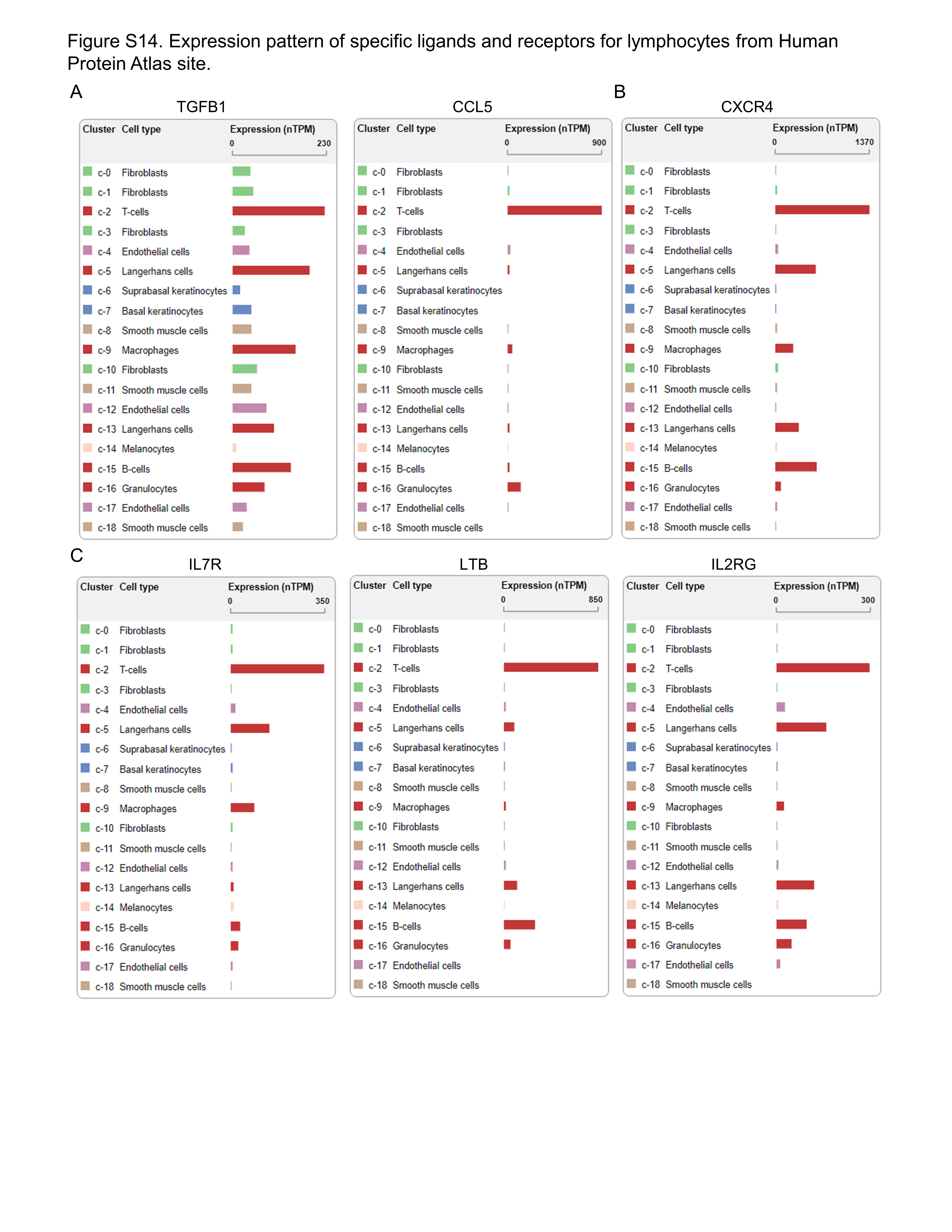

Supplement: Supplementary file 7 — Supplementary Material 7 [file 12964_2024_1725_MOESM7_ESM.jpg]

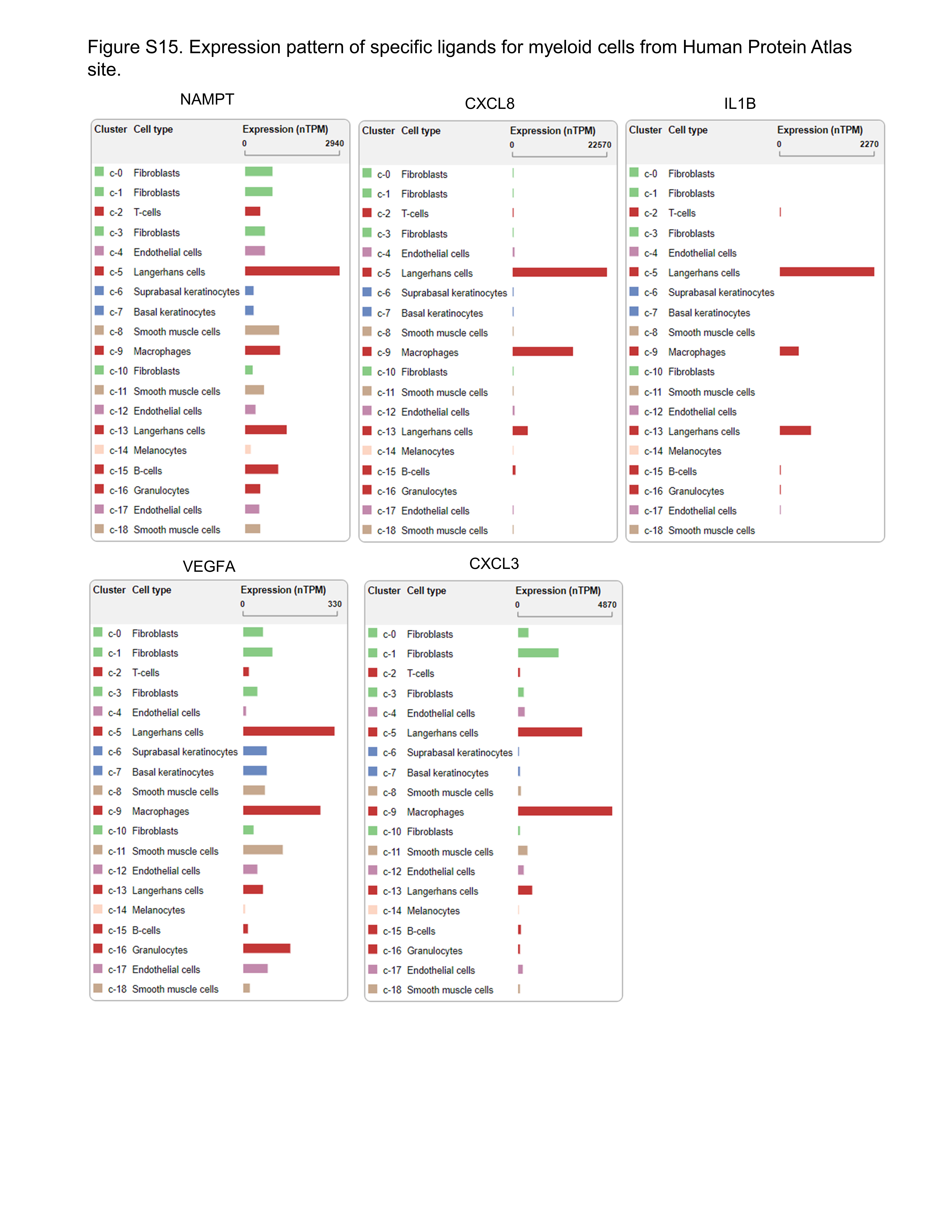

Supplement: Supplementary file 8 — Supplementary Material 8 [file 12964_2024_1725_MOESM8_ESM.jpg]

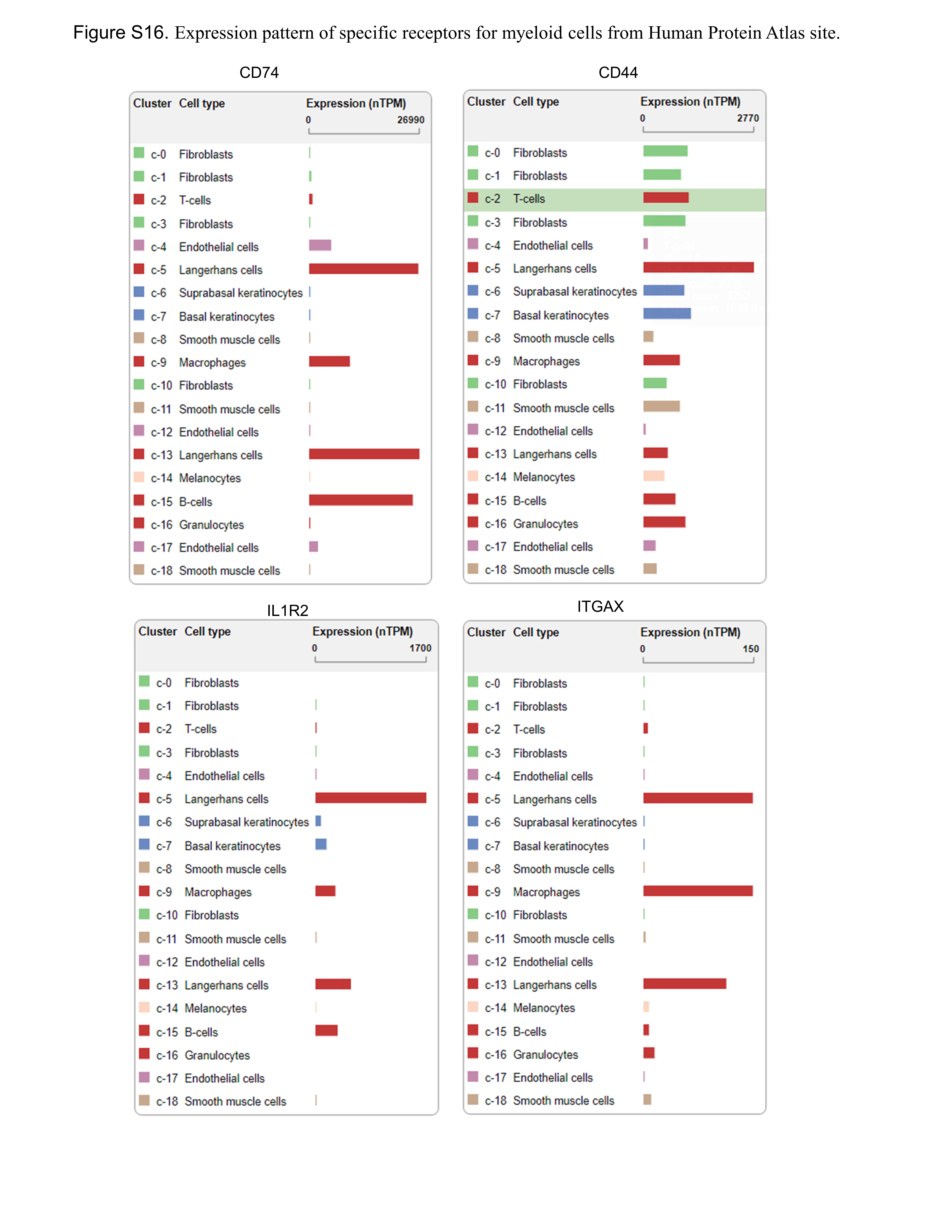

Supplement: Supplementary file 9 — Supplementary Material 9 [file 12964_2024_1725_MOESM9_ESM.jpg]

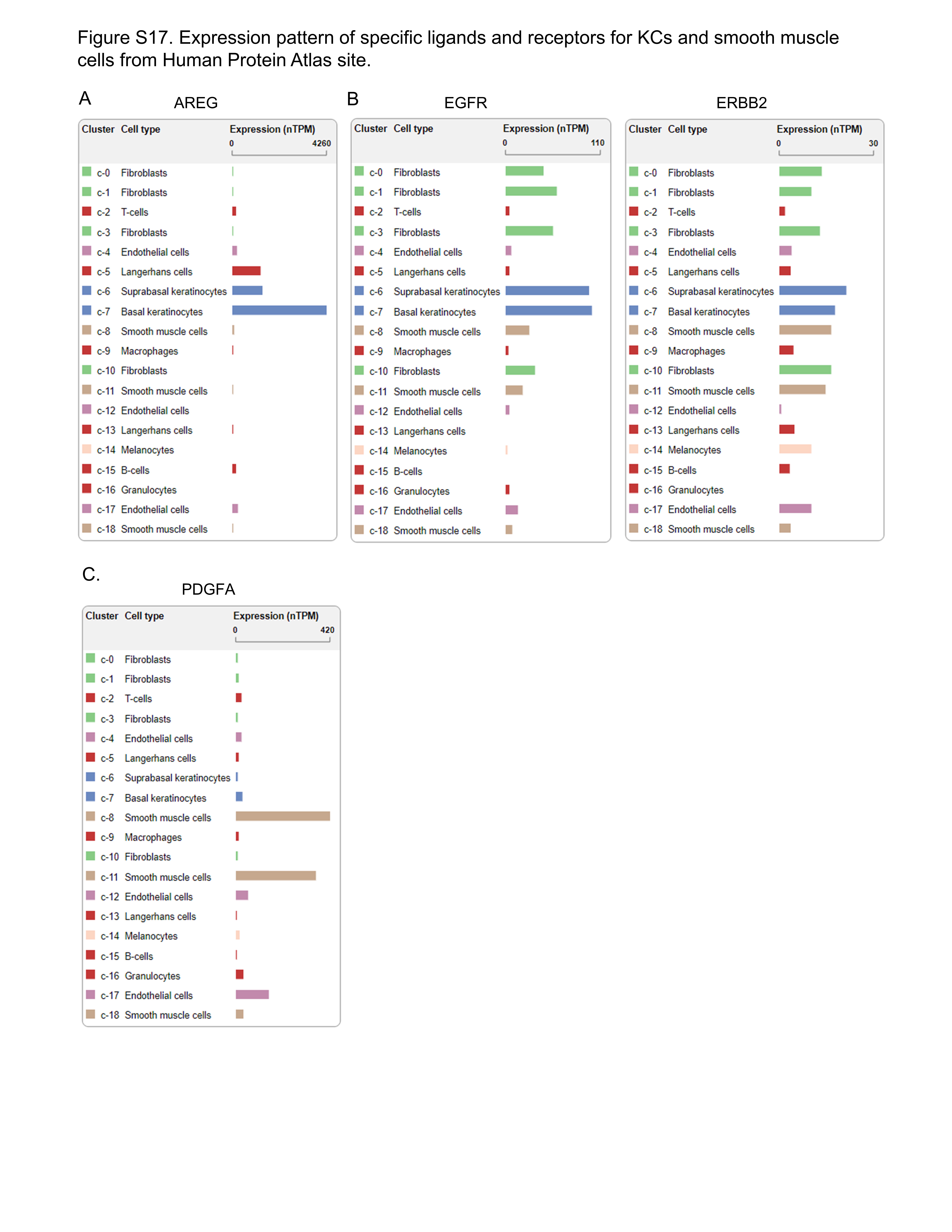

Supplement: Supplementary file 10 — Supplementary Material 10 [file 12964_2024_1725_MOESM10_ESM.jpg]

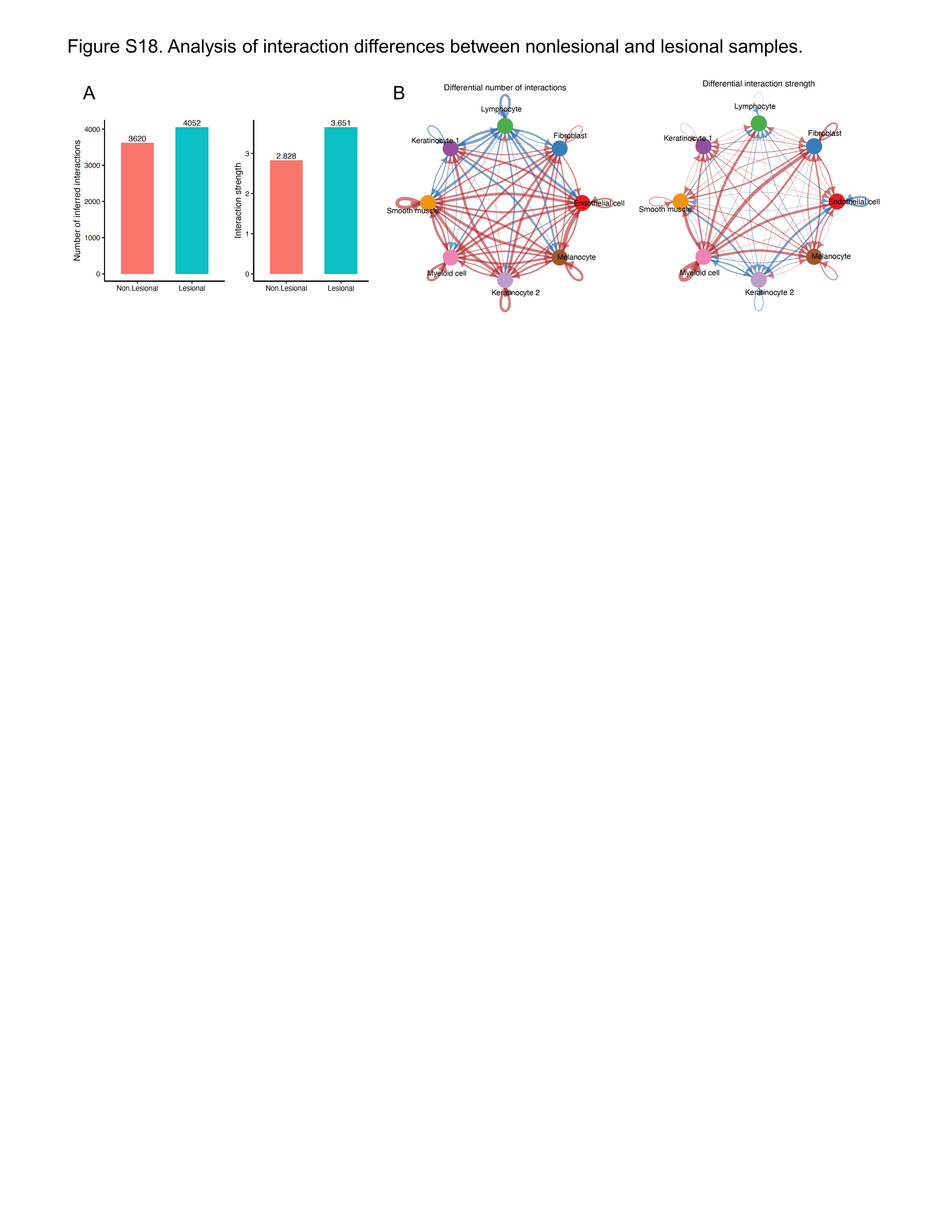

Supplement: Supplementary file 11 — Supplementary Material 11 [file 12964_2024_1725_MOESM11_ESM.jpg]

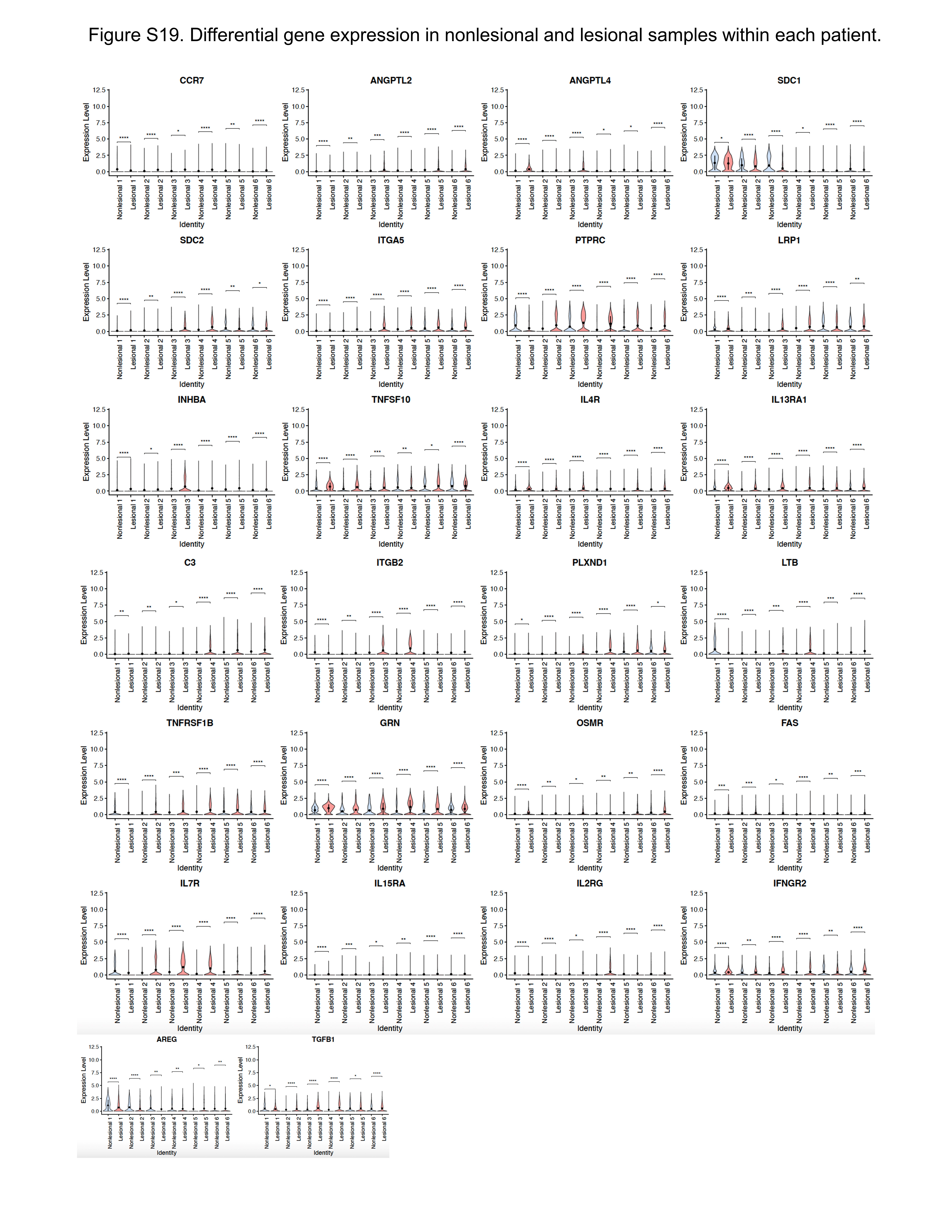

Supplement: Supplementary file 12 — Supplementary Material 12 [file 12964_2024_1725_MOESM12_ESM.jpg]

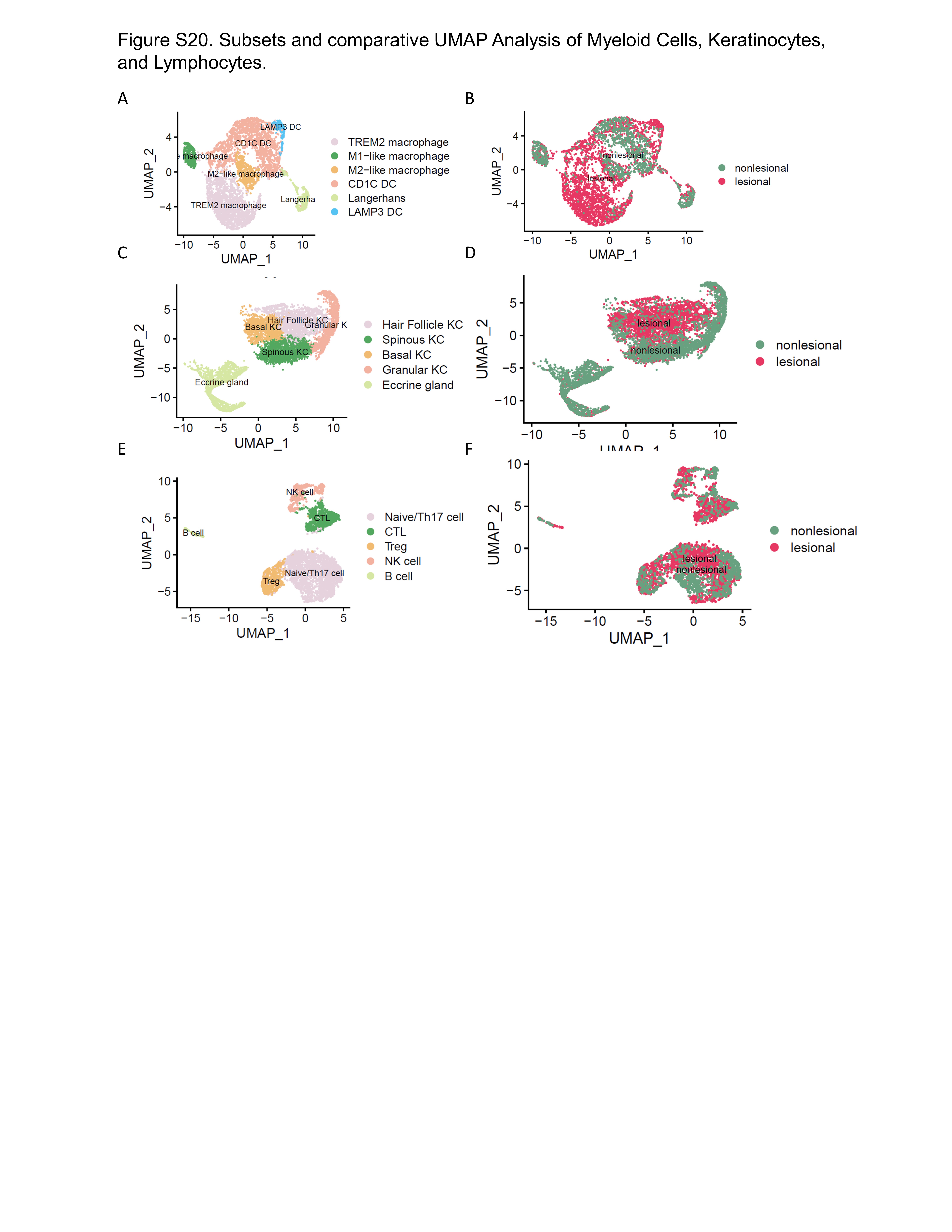

Supplement: Supplementary file 13 — Supplementary Material 13 [file 12964_2024_1725_MOESM13_ESM.jpg]

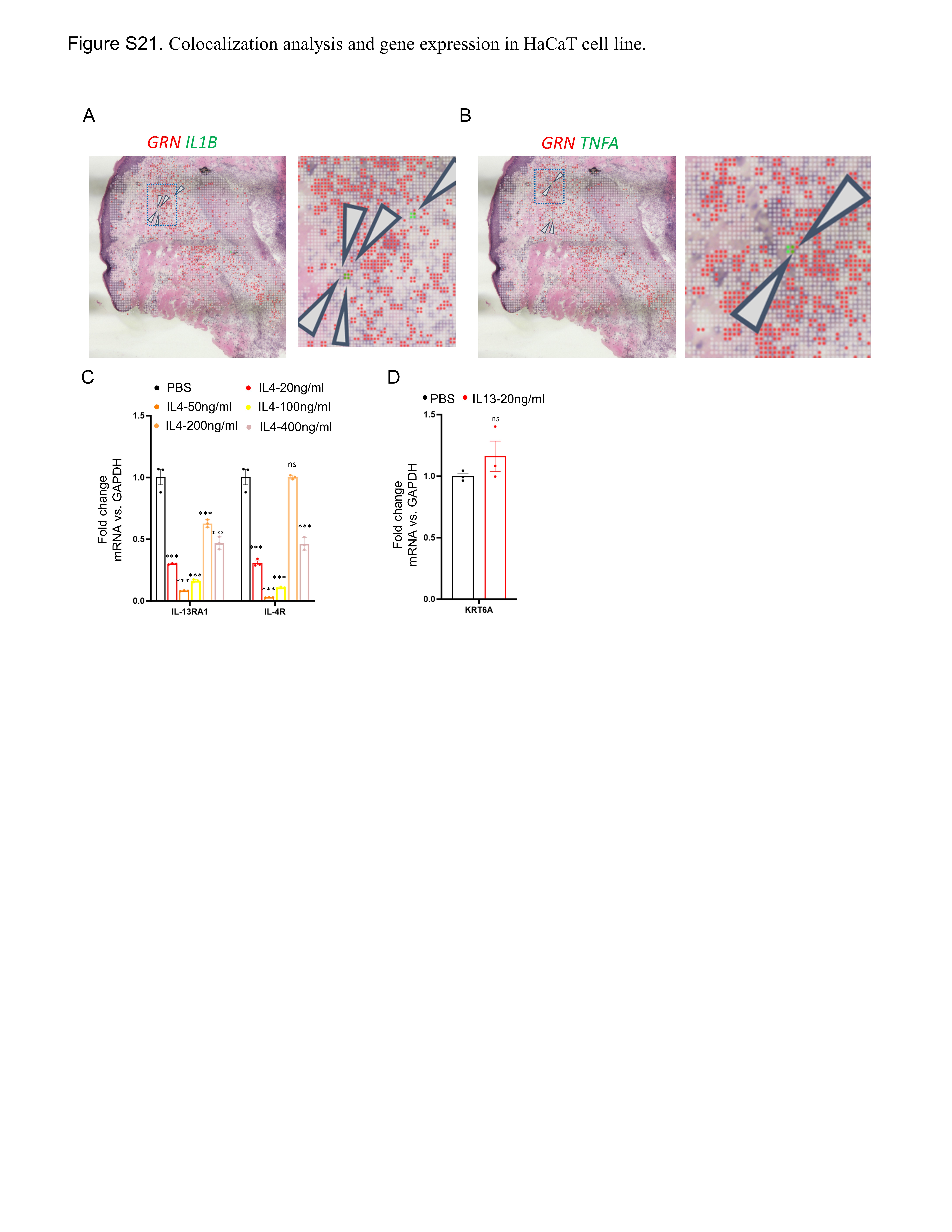

Supplement: Supplementary file 14 — Supplementary Material 14 [file 12964_2024_1725_MOESM14_ESM.jpg]
