# Supplementary material for: Analysis of intracellular communication reveals consistent gene changes associated with early-stage acne skin
Source: Cell Commun Signal. 2024 Aug 14;22:400. doi: 10.1186/s12964-024-01725-4 (PMC11325718; doi:10.1186/s12964-024-01725-4)
Supplement: Supplementary file 2 — Supplementary Material 2 [file 12964_2024_1725_MOESM2_ESM.pdf]

Fig S2

Outgoing signal from endothelial cell

Incoming signal to endothelial cell

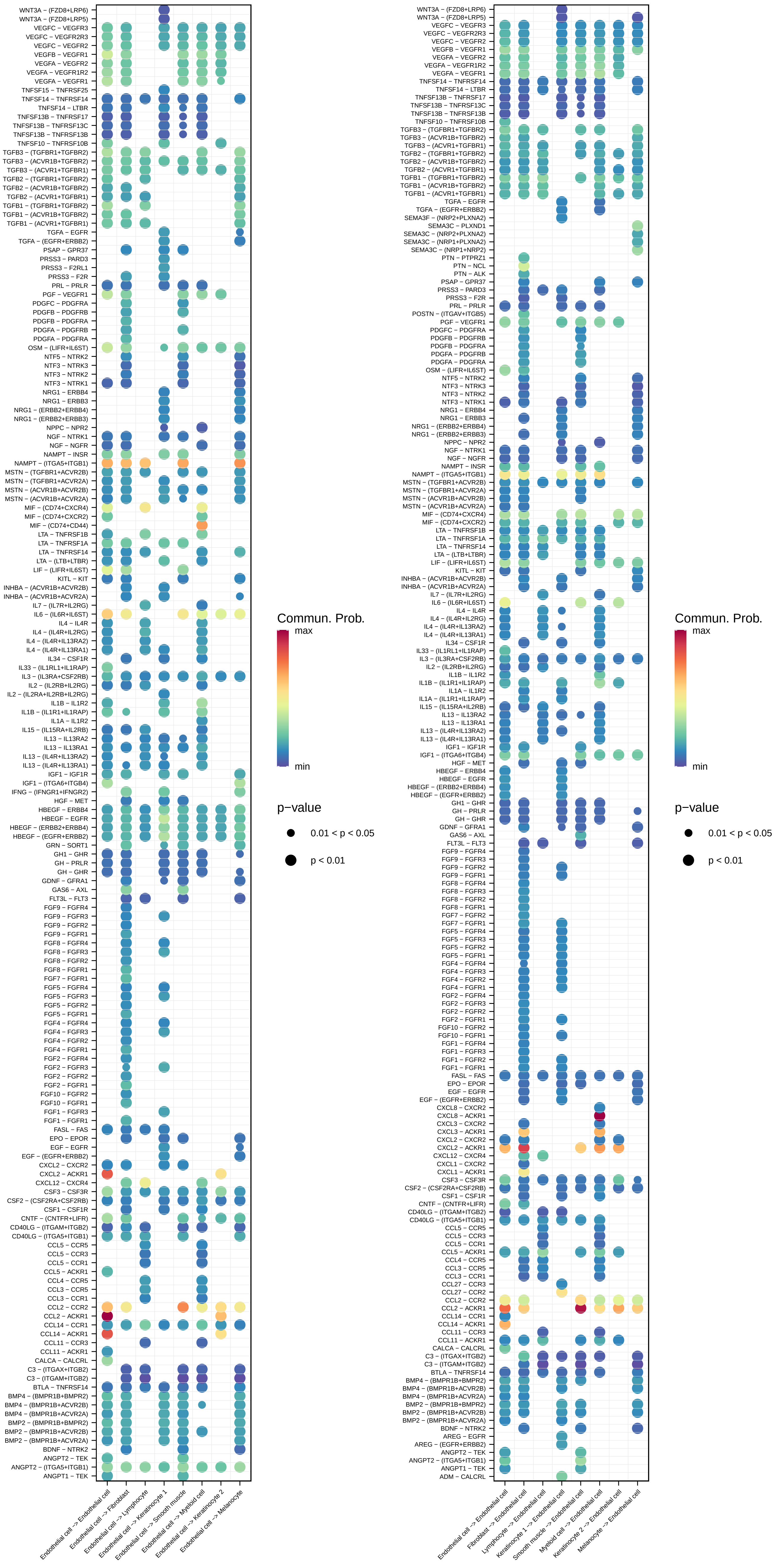

Fig S3

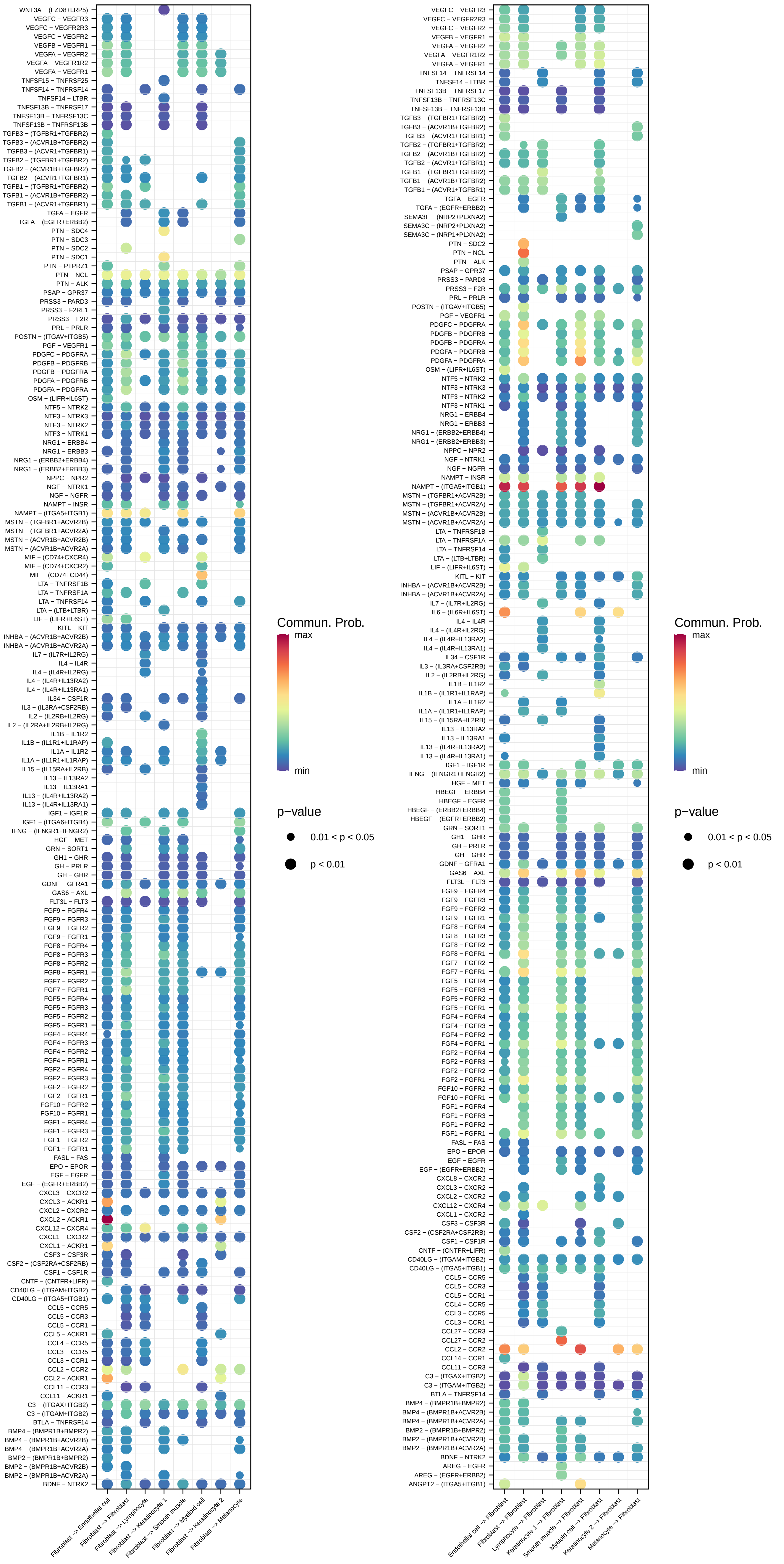

Outgoing signal from fibroblast
Incoming signal to fibroblast
Fibroblast -> Endothelial cell
Fibroblast -> Fibroblast
Fibroblast -> Lymphocyte
Fibroblast -> Keratinocyte 1
Fibroblast -> Smooth muscle
Fibroblast -> Myeloid cell
Fibroblast -> Keratinocyte 2
Fibroblast -> Melanocyte
Endothelial cell -> Fibroblast
Fibroblast -> Fibroblast
Lymphocyte -> Fibroblast
Keratinocyte 1 -> Fibroblast
Smooth muscle -> Fibroblast
Myeloid cell -> Fibroblast
Keratinocyte 2 -> Fibroblast
Melanocyte -> Fibroblast
Commun. Prob.
max
min
p-value
0.01 < p < 0.05
p < 0.01

Fig S4

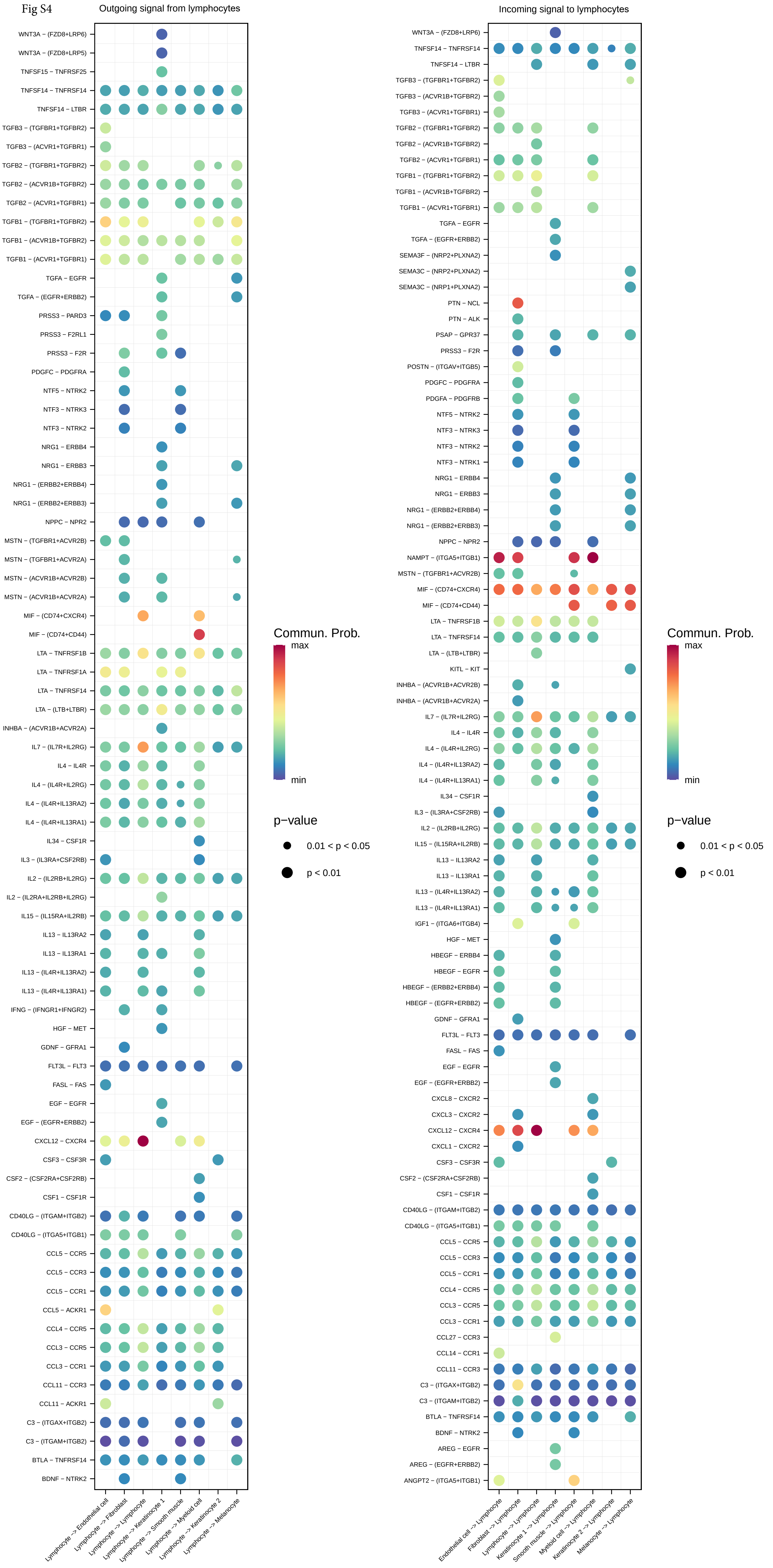

Outgoing signal from lymphocytes
WNT3A − (FZD8+LRP6)
WNT3A − (FZD8+LRP5)
TNFSF15 − TNFRSF25
TNFSF14 − TNFRSF14
TNFSF14 − LTBR
TGFB3 − (TGFBR1+TGFBR2)
TGFB3 − (ACVR1+TGFBR1)
TGFB2 − (TGFBR1+TGFBR2)
TGFB2 − (ACVR1B+TGFBR2)
TGFB2 − (ACVR1+TGFBR1)
TGFB1 − (TGFBR1+TGFBR2)
TGFB1 − (ACVR1B+TGFBR2)
TGFB1 − (ACVR1+TGFBR1)
TGFA − EGFR
TGFA − (EGFR+ERBB2)
PRSS3 − PARD3
PRSS3 − F2RL1
PRSS3 − F2R
PDGFC − PDGFRA
NTF5 − NTRK2
NTF3 − NTRK3
NTF3 − NTRK2
NRG1 − ERBB4
NRG1 − ERBB3
NRG1 − (ERBB2+ERBB4)
NRG1 − (ERBB2+ERBB3)
NPPC − NPR2
MSTN − (TGFBR1+ACVR2B)
MSTN − (TGFBR1+ACVR2A)
MSTN − (ACVR1B+ACVR2B)
MSTN − (ACVR1B+ACVR2A)
MIF − (CD74+CXCR4)
MIF − (CD74+CD44)
LTA − TNFRSF1B
LTA − TNFRSF1A
LTA − TNFRSF14
LTA − (LTB+LTBR)
INHBA − (ACVR1B+ACVR2A)
IL7 − (IL7R+IL2RG)
IL4 − IL4R
IL4 − (IL4R+IL2RG)
IL4 − (IL4R+IL13RA2)
IL4 − (IL4R+IL13RA1)
IL34 − CSF1R
IL3 − (IL3RA+CSF2RB)
IL2 − (IL2RB+IL2RG)
IL2 − (IL2RA+IL2RB+IL2RG)
IL15 − (IL15RA+IL2RB)
IL13 − IL13RA2
IL13 − IL13RA1
IL13 − (IL4R+IL13RA2)
IL13 − (IL4R+IL13RA1)
IFNG − (IFNGR1+IFNGR2)
HGF − MET
GDNF − GFRA1
FLT3L − FLT3
FASL − FAS
EGF − EGFR
EGF − (EGFR+ERBB2)
CXCL12 − CXCR4
CSF3 − CSF3R
CSF2 − (CSF2RA+CSF2RB)
CSF1 − CSF1R
CD40LG − (ITGAM+ITGB2)
CD40LG − (ITGA5+ITGB1)
CCL5 − CCR5
CCL5 − CCR3
CCL5 − CCR1
CCL5 − ACKR1
CCL4 − CCR5
CCL3 − CCR5
CCL3 − CCR1
CCL11 − CCR3
CCL11 − ACKR1
C3 − (ITGAX+ITGB2)
C3 − (ITGAM+ITGB2)
BTLA − TNFRSF14
BDNF − NTRK2
Lymphocyte −> Endothelial cell
Lymphocyte −> Fibroblast
Lymphocyte −> Lymphocyte
Lymphocyte −> Keratinocyte 1
Lymphocyte −> Smooth muscle
Lymphocyte −> Myeloid cell
Lymphocyte −> Keratinocyte 2
Lymphocyte −> Melanocyte
Commun. Prob.
max
min
p−value
0.01 < p < 0.05
p < 0.01
Incoming signal to lymphocytes
WNT3A − (FZD8+LRP6)
TNFSF14 − TNFRSF14
TNFSF14 − LTBR
TGFB3 − (TGFBR1+TGFBR2)
TGFB3 − (ACVR1B+TGFBR2)
TGFB3 − (ACVR1+TGFBR1)
TGFB2 − (TGFBR1+TGFBR2)
TGFB2 − (ACVR1B+TGFBR2)
TGFB2 − (ACVR1+TGFBR1)
TGFB1 − (TGFBR1+TGFBR2)
TGFB1 − (ACVR1B+TGFBR2)
TGFB1 − (ACVR1+TGFBR1)
TGFA − EGFR
TGFA − (EGFR+ERBB2)
SEMA3F − (NRP2+PLXNA2)
SEMA3C − (NRP2+PLXNA2)
SEMA3C − (NRP1+PLXNA2)
PTN − NCL
PTN − ALK
PSAP − GPR37
PRSS3 − F2R
POSTN − (ITGAV+ITGB5)
PDGFC − PDGFRA
PDGFA − PDGFRB
NTF5 − NTRK2
NTF3 − NTRK3
NTF3 − NTRK2
NTF3 − NTRK1
NRG1 − ERBB4
NRG1 − ERBB3
NRG1 − (ERBB2+ERBB4)
NRG1 − (ERBB2+ERBB3)
NPPC − NPR2
NAMPT − (ITGA5+ITGB1)
MSTN − (TGFBR1+ACVR2B)
MIF − (CD74+CXCR4)
MIF − (CD74+CD44)
LTA − TNFRSF1B
LTA − TNFRSF14
LTA − (LTB+LTBR)
KITL − KIT
INHBA − (ACVR1B+ACVR2B)
INHBA − (ACVR1B+ACVR2A)
IL7 − (IL7R+IL2RG)
IL4 − IL4R
IL4 − (IL4R+IL2RG)
IL4 − (IL4R+IL13RA2)
IL4 − (IL4R+IL13RA1)
IL34 − CSF1R
IL3 − (IL3RA+CSF2RB)
IL2 − (IL2RB+IL2RG)
IL15 − (IL15RA+IL2RB)
IL13 − IL13RA2
IL13 − IL13RA1
IL13 − (IL4R+IL13RA2)
IL13 − (IL4R+IL13RA1)
IGF1 − (ITGA6+ITGB4)
HGF − MET
HBEGF − ERBB4
HBEGF − EGFR
HBEGF − (ERBB2+ERBB4)
HBEGF − (EGFR+ERBB2)
GDNF − GFRA1
FLT3L − FLT3
FASL − FAS
EGF − EGFR
EGF − (EGFR+ERBB2)
CXCL8 − CXCR2
CXCL3 − CXCR2
CXCL12 − CXCR4
CXCL1 − CXCR2
CSF3 − CSF3R
CSF2 − (CSF2RA+CSF2RB)
CSF1 − CSF1R
CD40LG − (ITGAM+ITGB2)
CD40LG − (ITGA5+ITGB1)
CCL5 − CCR5
CCL5 − CCR3
CCL5 − CCR1
CCL4 − CCR5
CCL3 − CCR5
CCL3 − CCR1
CCL27 − CCR3
CCL14 − CCR1
CCL11 − CCR3
C3 − (ITGAX+ITGB2)
C3 − (ITGAM+ITGB2)
BTLA − TNFRSF14
BDNF − NTRK2
AREG − EGFR
AREG − (EGFR+ERBB2)
ANGPT2 − (ITGA5+ITGB1)
Endothelial cell −> Lymphocyte
Fibroblast −> Lymphocyte
Lymphocyte −> Lymphocyte
Keratinocyte 1 −> Lymphocyte
Smooth muscle −> Lymphocyte
Myeloid cell −> Lymphocyte
Keratinocyte 2 −> Lymphocyte
Melanocyte −> Lymphocyte
Commun. Prob.
max
min
p−value
0.01 < p < 0.05
p < 0.01

Fig S5

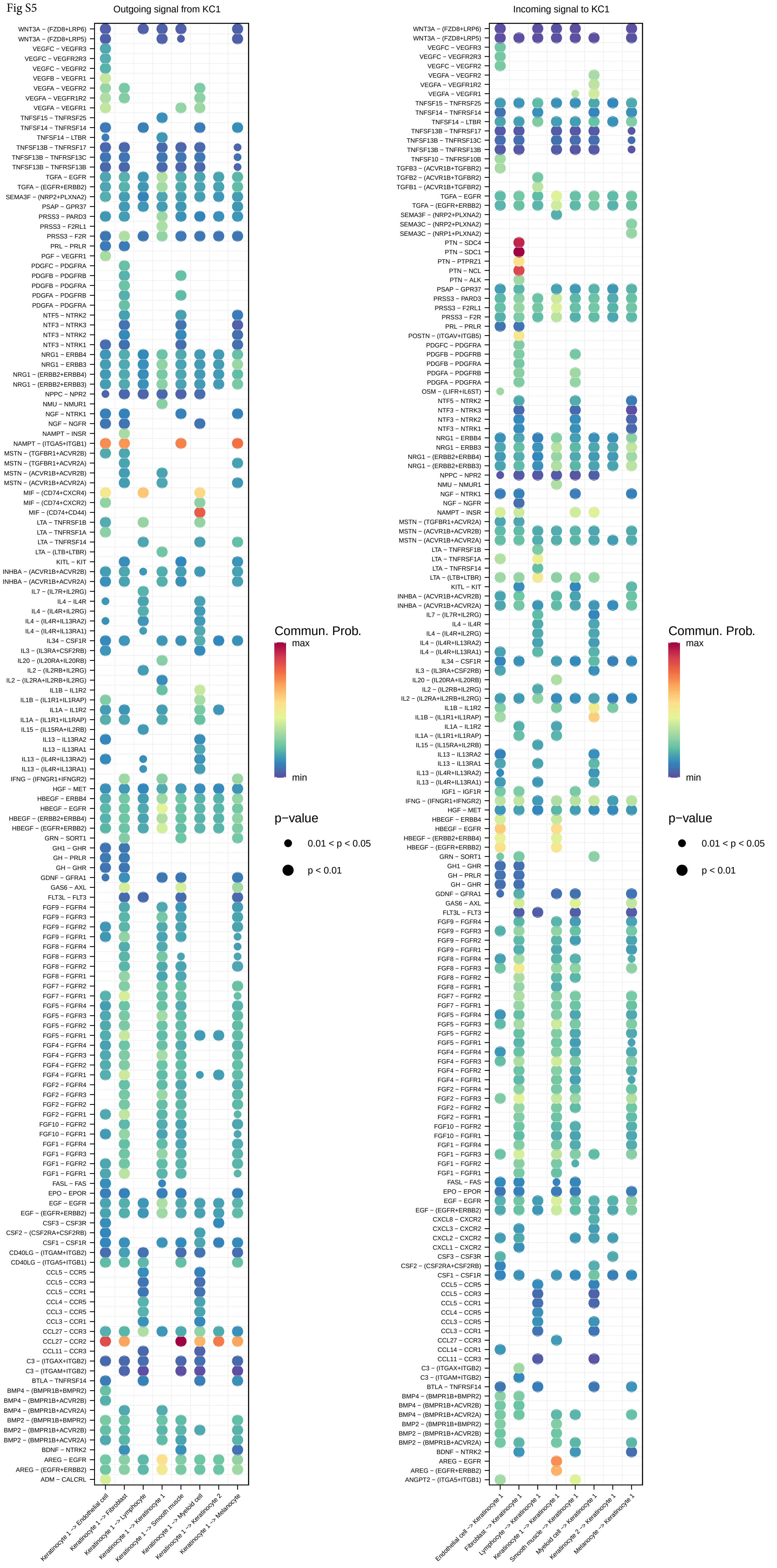
Outgoing signal from KC1
Incoming signal to KC1
Commun. Prob.
max
min
p−value
0.01 < p < 0.05
p < 0.01
Keratinocyte 1 −> Endothelial cell
Keratinocyte 1 −> Fibroblast
Keratinocyte 1 −> Lymphocyte
Keratinocyte 1 −> Keratinocyte 1
Keratinocyte 1 −> Smooth muscle
Keratinocyte 1 −> Myeloid cell
Keratinocyte 1 −> Keratinocyte 2
Keratinocyte 1 −> Melanocyte
Endothelial cell −> Keratinocyte 1
Fibroblast −> Keratinocyte 1
Lymphocyte −> Keratinocyte 1
Keratinocyte 1 −> Keratinocyte 1
Smooth muscle −> Keratinocyte 1
Myeloid cell −> Keratinocyte 1
Keratinocyte 2 −> Keratinocyte 1
Melanocyte −> Keratinocyte 1

Fig S6

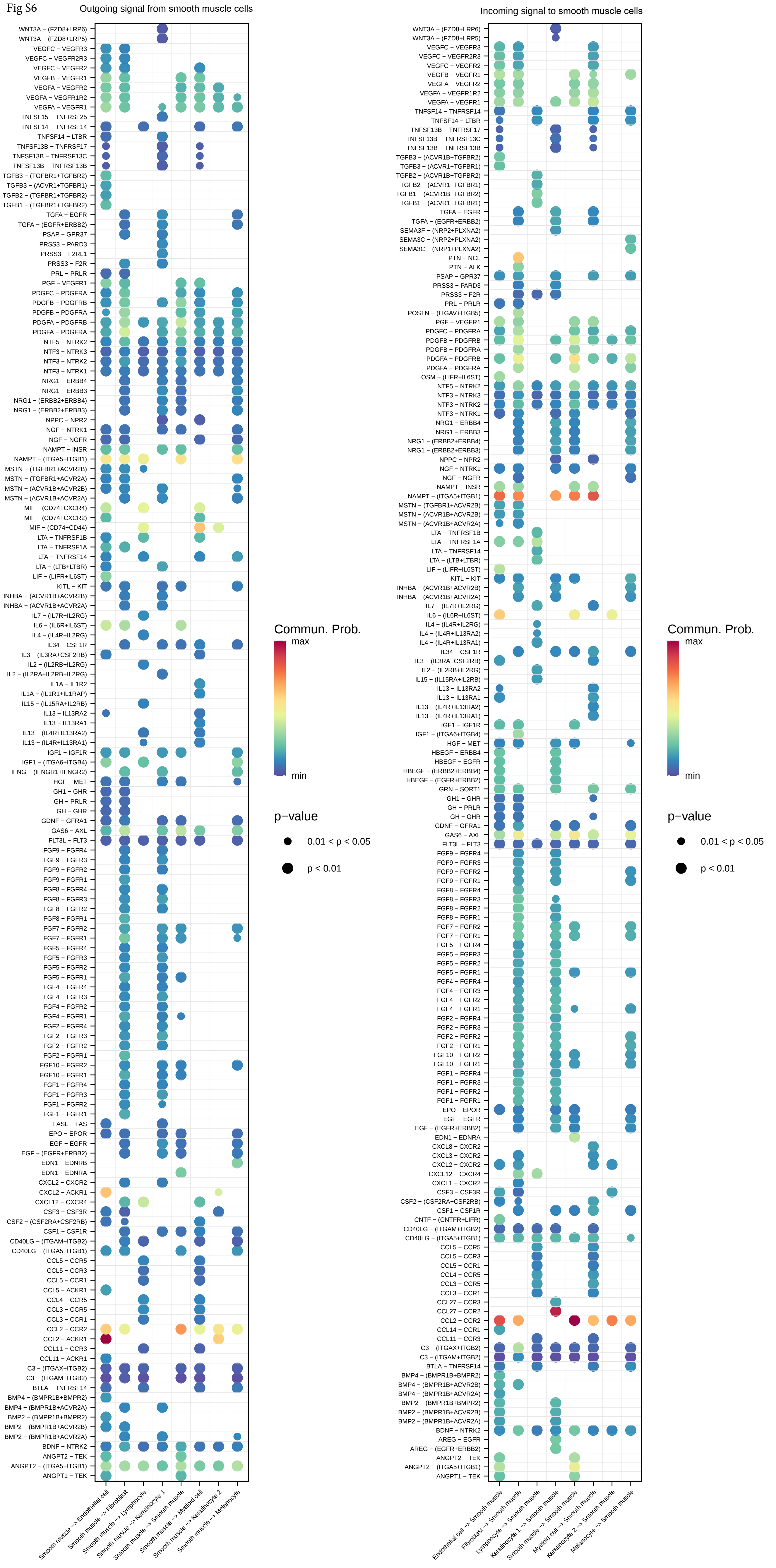
Outgoing signal from smooth muscle cells
WNT3A – (FZD8+LRP6)
WNT3A – (FZD8+LRP5)
VEGFC – VEGFR3
VEGFC – VEGFR2R3
VEGFC – VEGFR2
VEGFB – VEGFR1
VEGFA – VEGFR2
VEGFA – VEGFR1R2
VEGFA – VEGFR1
TNFSF15 – TNFRSF25
TNFSF14 – TNFRSF14
TNFSF14 – LTBR
TNFSF13B – TNFRSF17
TNFSF13B – TNFRSF13C
TNFSF13B – TNFRSF13B
TGFB3 – (TGFBR1+TGFBR2)
TGFB3 – (ACVR1+TGFBR1)
TGFB2 – (TGFBR1+TGFBR2)
TGFB1 – (TGFBR1+TGFBR2)
TGFA – EGFR
TGFA – (EGFR+ERBB2)
PSAP – GPR37
PRSS3 – PARD3
PRSS3 – F2RL1
PRSS3 – F2R
PRL – PRLR
PGF – VEGFR1
PDGFC – PDGFRA
PDGFB – PDGFRB
PDGFB – PDGFRA
PDGFA – PDGFRB
PDGFA – PDGFRA
NTF5 – NTRK2
NTF3 – NTRK3
NTF3 – NTRK2
NTF3 – NTRK1
NRG1 – ERBB4
NRG1 – ERBB3
NRG1 – (ERBB2+ERBB4)
NRG1 – (ERBB2+ERBB3)
NPPC – NPR2
NGF – NTRK1
NGF – NGFR
NAMPT – INSR
NAMPT – (ITGA5+ITGB1)
MSTN – (TGFBR1+ACVR2B)
MSTN – (TGFBR1+ACVR2A)
MSTN – (ACVR1B+ACVR2B)
MSTN – (ACVR1B+ACVR2A)
MIF – (CD74+CXCR4)
MIF – (CD74+CXCR2)
MIF – (CD74+CD44)
LTA – TNFRSF1B
LTA – TNFRSF1A
LTA – TNFRSF14
LTA – (LTB+LTBR)
LIF – (LIFR+IL6ST)
KITL – KIT
INHBA – (ACVR1B+ACVR2B)
INHBA – (ACVR1B+ACVR2A)
IL7 – (IL7R+IL2RG)
IL6 – (IL6R+IL6ST)
IL4 – (IL4R+IL2RG)
IL34 – CSF1R
IL3 – (IL3RA+CSF2RB)
IL2 – (IL2RB+IL2RG)
IL2 – (IL2RA+IL2RB+IL2RG)
IL1A – IL1R2
IL1A – (IL1R1+IL1RAP)
IL15 – (IL15RA+IL2RB)
IL13 – IL13RA2
IL13 – IL13RA1
IL13 – (IL4R+IL13RA2)
IL13 – (IL4R+IL13RA1)
IGF1 – IGF1R
IGF1 – (ITGA6+ITGB4)
IFNG – (IFNGR1+IFNGR2)
HGF – MET
GH1 – GHR
GH – PRLR
GH – GHR
GDNF – GFRA1
GAS6 – AXL
FLT3L – FLT3
FGF9 – FGFR4
FGF9 – FGFR3
FGF9 – FGFR2
FGF9 – FGFR1
FGF8 – FGFR4
FGF8 – FGFR3
FGF8 – FGFR2
FGF8 – FGFR1
FGF7 – FGFR2
FGF7 – FGFR1
FGF5 – FGFR4
FGF5 – FGFR3
FGF5 – FGFR2
FGF5 – FGFR1
FGF4 – FGFR4
FGF4 – FGFR3
FGF4 – FGFR2
FGF4 – FGFR1
FGF2 – FGFR4
FGF2 – FGFR3
FGF2 – FGFR2
FGF2 – FGFR1
FGF10 – FGFR2
FGF10 – FGFR1
FGF1 – FGFR4
FGF1 – FGFR3
FGF1 – FGFR2
FGF1 – FGFR1
FASL – FAS
EPO – EPOR
EGF – EGFR
EGF – (EGFR+ERBB2)
EDN1 – EDNRB
EDN1 – EDNRA
CXCL2 – CXCR2
CXCL2 – ACKR1
CXCL12 – CXCR4
CSF3 – CSF3R
CSF2 – (CSF2RA+CSF2RB)
CSF1 – CSF1R
CD40LG – (ITGAM+ITGB2)
CD40LG – (ITGA5+ITGB1)
CCL5 – CCR5
CCL5 – CCR3
CCL5 – CCR1
CCL5 – ACKR1
CCL4 – CCR5
CCL3 – CCR5
CCL3 – CCR1
CCL2 – CCR2
CCL2 – ACKR1
CCL11 – CCR3
CCL11 – ACKR1
C3 – (ITGAX+ITGB2)
C3 – (ITGAM+ITGB2)
BTLA – TNFRSF14
BMP4 – (BMPR1B+BMPR2)
BMP4 – (BMPR1B+ACVR2A)
BMP2 – (BMPR1B+BMPR2)
BMP2 – (BMPR1B+ACVR2B)
BMP2 – (BMPR1B+ACVR2A)
BDNF – NTRK2
ANGPT2 – TEK
ANGPT2 – (ITGA5+ITGB1)
ANGPT1 – TEK
Smooth muscle -> Endothelial cell
Smooth muscle -> Fibroblast
Smooth muscle -> Lymphocyte
Smooth muscle -> Keratinocyte 1
Smooth muscle -> Smooth muscle
Smooth muscle -> Myeloid cell
Smooth muscle -> Keratinocyte 2
Smooth muscle -> Melanocyte
Commun. Prob.
max
min
p−value
0.01 < p < 0.05
p < 0.01
Incoming signal to smooth muscle cells
WNT3A – (FZD8+LRP6)
WNT3A – (FZD8+LRP5)
VEGFC – VEGFR3
VEGFC – VEGFR2R3
VEGFC – VEGFR2
VEGFB – VEGFR1
VEGFA – VEGFR2
VEGFA – VEGFR1R2
VEGFA – VEGFR1
TNFSF14 – TNFRSF14
TNFSF14 – LTBR
TNFSF13B – TNFRSF17
TNFSF13B – TNFRSF13C
TNFSF13B – TNFRSF13B
TGFB3 – (ACVR1B+TGFBR2)
TGFB3 – (ACVR1+TGFBR1)
TGFB2 – (ACVR1B+TGFBR2)
TGFB2 – (ACVR1+TGFBR1)
TGFB1 – (ACVR1B+TGFBR2)
TGFB1 – (ACVR1+TGFBR1)
TGFA – EGFR
TGFA – (EGFR+ERBB2)
SEMA3F – (NRP2+PLXNA2)
SEMA3C – (NRP2+PLXNA2)
SEMA3C – (NRP1+PLXNA2)
PTN – NCL
PTN – ALK
PSAP – GPR37
PRSS3 – PARD3
PRSS3 – F2R
PRL – PRLR
POSTN – (ITGAV+ITGB5)
PGF – VEGFR1
PDGFC – PDGFRA
PDGFB – PDGFRB
PDGFB – PDGFRA
PDGFA – PDGFRB
PDGFA – PDGFRA
OSM – (LIFR+IL6ST)
NTF5 – NTRK2
NTF3 – NTRK3
NTF3 – NTRK2
NTF3 – NTRK1
NRG1 – ERBB4
NRG1 – ERBB3
NRG1 – (ERBB2+ERBB4)
NRG1 – (ERBB2+ERBB3)
NPPC – NPR2
NGF – NTRK1
NGF – NGFR
NAMPT – INSR
NAMPT – (ITGA5+ITGB1)
MSTN – (TGFBR1+ACVR2B)
MSTN – (ACVR1B+ACVR2B)
MSTN – (ACVR1B+ACVR2A)
LTA – TNFRSF1B
LTA – TNFRSF1A
LTA – TNFRSF14
LTA – (LTB+LTBR)
LIF – (LIFR+IL6ST)
KITL – KIT
INHBA – (ACVR1B+ACVR2B)
INHBA – (ACVR1B+ACVR2A)
IL7 – (IL7R+IL2RG)
IL6 – (IL6R+IL6ST)
IL4 – (IL4R+IL2RG)
IL4 – (IL4R+IL13RA2)
IL4 – (IL4R+IL13RA1)
IL34 – CSF1R
IL3 – (IL3RA+CSF2RB)
IL2 – (IL2RB+IL2RG)
IL15 – (IL15RA+IL2RB)
IL13 – IL13RA2
IL13 – IL13RA1
IL13 – (IL4R+IL13RA2)
IL13 – (IL4R+IL13RA1)
IGF1 – IGF1R
IGF1 – (ITGA6+ITGB4)
HGF – MET
HBEGF – ERBB4
HBEGF – EGFR
HBEGF – (ERBB2+ERBB4)
HBEGF – (EGFR+ERBB2)
GRN – SORT1
GH1 – GHR
GH – PRLR
GH – GHR
GDNF – GFRA1
GAS6 – AXL
FLT3L – FLT3
FGF9 – FGFR4
FGF9 – FGFR3
FGF9 – FGFR2
FGF9 – FGFR1
FGF8 – FGFR4
FGF8 – FGFR3
FGF8 – FGFR2
FGF8 – FGFR1
FGF7 – FGFR2
FGF7 – FGFR1
FGF5 – FGFR4
FGF5 – FGFR3
FGF5 – FGFR2
FGF5 – FGFR1
FGF4 – FGFR4
FGF4 – FGFR3
FGF4 – FGFR2
FGF4 – FGFR1
FGF2 – FGFR4
FGF2 – FGFR3
FGF2 – FGFR2
FGF2 – FGFR1
FGF10 – FGFR2
FGF10 – FGFR1
FGF1 – FGFR4
FGF1 – FGFR3
FGF1 – FGFR2
FGF1 – FGFR1
EPO – EPOR
EGF – EGFR
EGF – (EGFR+ERBB2)
EDN1 – EDNRA
CXCL8 – CXCR2
CXCL3 – CXCR2
CXCL2 – CXCR2
CXCL12 – CXCR4
CXCL1 – CXCR2
CSF3 – CSF3R
CSF2 – (CSF2RA+CSF2RB)
CSF1 – CSF1R
CNTF – (CNTFR+LIFR)
CD40LG – (ITGAM+ITGB2)
CD40LG – (ITGA5+ITGB1)
CCL5 – CCR5
CCL5 – CCR3
CCL5 – CCR1
CCL4 – CCR5
CCL3 – CCR5
CCL3 – CCR1
CCL27 – CCR3
CCL27 – CCR2
CCL2 – CCR2
CCL14 – CCR1
CCL11 – CCR3
C3 – (ITGAX+ITGB2)
C3 – (ITGAM+ITGB2)
BTLA – TNFRSF14
BMP4 – (BMPR1B+BMPR2)
BMP4 – (BMPR1B+ACVR2B)
BMP4 – (BMPR1B+ACVR2A)
BMP2 – (BMPR1B+BMPR2)
BMP2 – (BMPR1B+ACVR2B)
BMP2 – (BMPR1B+ACVR2A)
BDNF – NTRK2
AREG – EGFR
AREG – (EGFR+ERBB2)
ANGPT2 – TEK
ANGPT2 – (ITGA5+ITGB1)
ANGPT1 – TEK
Endothelial cell -> Smooth muscle
Fibroblast -> Smooth muscle
Lymphocyte -> Smooth muscle
Keratinocyte 1 -> Smooth muscle
Smooth muscle -> Smooth muscle
Myeloid cell -> Smooth muscle
Keratinocyte 2 -> Smooth muscle
Melanocyte -> Smooth muscle
Commun. Prob.
max
min
p−value
0.01 < p < 0.05
p < 0.01

Fig S7

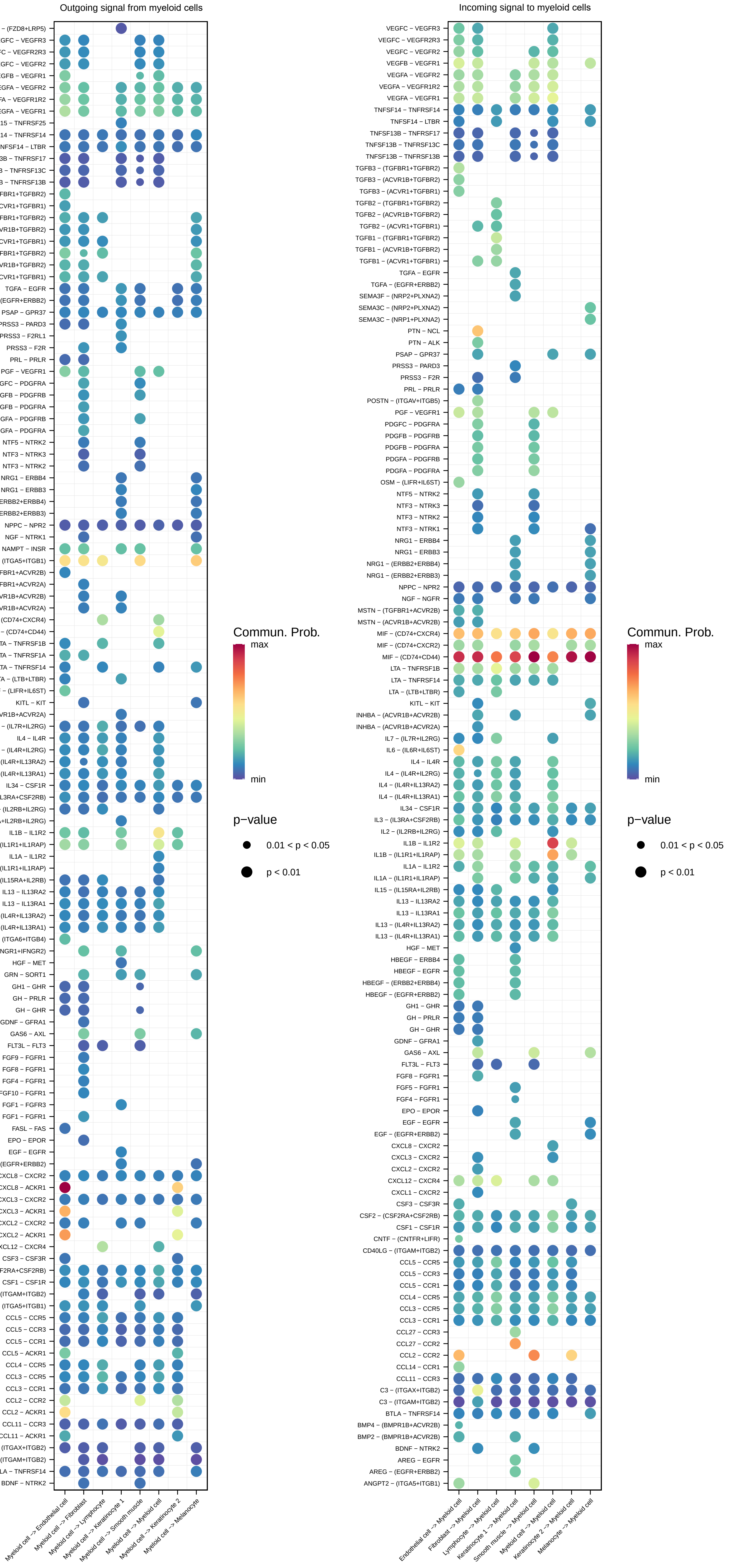

Outgoing signal from myeloid cells
WNT3A − (FZD8+LRP5)
VEGFC − VEGFR3
VEGFC − VEGFR2R3
VEGFC − VEGFR2
VEGFB − VEGFR1
VEGFA − VEGFR2
VEGFA − VEGFR1R2
VEGFA − VEGFR1
TNFSF15 − TNFRSF25
TNFSF14 − TNFRSF14
TNFSF14 − LTBR
TNFSF13B − TNFRSF17
TNFSF13B − TNFRSF13C
TNFSF13B − TNFRSF13B
TGFB3 − (TGFBR1+TGFBR2)
TGFB3 − (ACVR1+TGFBR1)
TGFB2 − (TGFBR1+TGFBR2)
TGFB2 − (ACVR1B+TGFBR2)
TGFB2 − (ACVR1+TGFBR1)
TGFB1 − (TGFBR1+TGFBR2)
TGFB1 − (ACVR1B+TGFBR2)
TGFB1 − (ACVR1+TGFBR1)
TGFA − EGFR
TGFA − (EGFR+ERBB2)
PSAP − GPR37
PRSS3 − PARD3
PRSS3 − F2RL1
PRSS3 − F2R
PRL − PRLR
PGF − VEGFR1
PDGFC − PDGFRA
PDGFB − PDGFRB
PDGFB − PDGFRA
PDGFA − PDGFRB
PDGFA − PDGFRA
NTF5 − NTRK2
NTF3 − NTRK3
NTF3 − NTRK2
NRG1 − ERBB4
NRG1 − ERBB3
NRG1 − (ERBB2+ERBB4)
NRG1 − (ERBB2+ERBB3)
NPPC − NPR2
NGF − NTRK1
NAMPT − INSR
NAMPT − (ITGA5+ITGB1)
MSTN − (TGFBR1+ACVR2B)
MSTN − (TGFBR1+ACVR2A)
MSTN − (ACVR1B+ACVR2B)
MSTN − (ACVR1B+ACVR2A)
MIF − (CD74+CXCR4)
MIF − (CD74+CD44)
LTA − TNFRSF1B
LTA − TNFRSF1A
LTA − TNFRSF14
LTA − (LTB+LTBR)
LIF − (LIFR+IL6ST)
KITL − KIT
INHBA − (ACVR1B+ACVR2A)
IL7 − (IL7R+IL2RG)
IL4 − IL4R
IL4 − (IL4R+IL2RG)
IL4 − (IL4R+IL13RA2)
IL4 − (IL4R+IL13RA1)
IL34 − CSF1R
IL3 − (IL3RA+CSF2RB)
IL2 − (IL2RB+IL2RG)
IL2 − (IL2RA+IL2RB+IL2RG)
IL1B − IL1R2
IL1B − (IL1R1+IL1RAP)
IL1A − IL1R2
IL1A − (IL1R1+IL1RAP)
IL15 − (IL15RA+IL2RB)
IL13 − IL13RA2
IL13 − IL13RA1
IL13 − (IL4R+IL13RA2)
IL13 − (IL4R+IL13RA1)
IGF1 − (ITGA6+ITGB4)
IFNG − (IFNGR1+IFNGR2)
HGF − MET
GRN − SORT1
GH1 − GHR
GH − PRLR
GH − GHR
GDNF − GFRA1
GAS6 − AXL
FLT3L − FLT3
FGF9 − FGFR1
FGF8 − FGFR1
FGF4 − FGFR1
FGF10 − FGFR1
FGF1 − FGFR3
FGF1 − FGFR1
FASL − FAS
EPO − EPOR
EGF − EGFR
EGF − (EGFR+ERBB2)
CXCL8 − CXCR2
CXCL8 − ACKR1
CXCL3 − CXCR2
CXCL3 − ACKR1
CXCL2 − CXCR2
CXCL2 − ACKR1
CXCL12 − CXCR4
CSF3 − CSF3R
CSF2 − (CSF2RA+CSF2RB)
CSF1 − CSF1R
CD40LG − (ITGAM+ITGB2)
CD40LG − (ITGA5+ITGB1)
CCL5 − CCR5
CCL5 − CCR3
CCL5 − CCR1
CCL5 − ACKR1
CCL4 − CCR5
CCL3 − CCR5
CCL3 − CCR1
CCL2 − CCR2
CCL2 − ACKR1
CCL11 − CCR3
CCL11 − ACKR1
C3 − (ITGAX+ITGB2)
C3 − (ITGAM+ITGB2)
BTLA − TNFRSF14
BDNF − NTRK2
Myeloid cell −> Endothelial cell
Myeloid cell −> Fibroblast
Myeloid cell −> Lymphocyte
Myeloid cell −> Keratinocyte 1
Myeloid cell −> Smooth muscle
Myeloid cell −> Myeloid cell
Myeloid cell −> Keratinocyte 2
Myeloid cell −> Melanocyte
Commun. Prob.
max
min
p−value
0.01 < p < 0.05
p < 0.01
Incoming signal to myeloid cells
VEGFC − VEGFR3
VEGFC − VEGFR2R3
VEGFC − VEGFR2
VEGFB − VEGFR1
VEGFA − VEGFR2
VEGFA − VEGFR1R2
VEGFA − VEGFR1
TNFSF14 − TNFRSF14
TNFSF14 − LTBR
TNFSF13B − TNFRSF17
TNFSF13B − TNFRSF13C
TNFSF13B − TNFRSF13B
TGFB3 − (TGFBR1+TGFBR2)
TGFB3 − (ACVR1B+TGFBR2)
TGFB3 − (ACVR1+TGFBR1)
TGFB2 − (TGFBR1+TGFBR2)
TGFB2 − (ACVR1B+TGFBR2)
TGFB2 − (ACVR1+TGFBR1)
TGFB1 − (TGFBR1+TGFBR2)
TGFB1 − (ACVR1B+TGFBR2)
TGFB1 − (ACVR1+TGFBR1)
TGFA − EGFR
TGFA − (EGFR+ERBB2)
SEMA3F − (NRP2+PLXNA2)
SEMA3C − (NRP2+PLXNA2)
SEMA3C − (NRP1+PLXNA2)
PTN − NCL
PTN − ALK
PSAP − GPR37
PRSS3 − PARD3
PRSS3 − F2R
PRL − PRLR
POSTN − (ITGAV+ITGB5)
PGF − VEGFR1
PDGFC − PDGFRA
PDGFB − PDGFRB
PDGFB − PDGFRA
PDGFA − PDGFRB
PDGFA − PDGFRA
OSM − (LIFR+IL6ST)
NTF5 − NTRK2
NTF3 − NTRK3
NTF3 − NTRK2
NTF3 − NTRK1
NRG1 − ERBB4
NRG1 − ERBB3
NRG1 − (ERBB2+ERBB4)
NRG1 − (ERBB2+ERBB3)
NPPC − NPR2
NGF − NGFR
MSTN − (TGFBR1+ACVR2B)
MSTN − (ACVR1B+ACVR2B)
MIF − (CD74+CXCR4)
MIF − (CD74+CXCR2)
MIF − (CD74+CD44)
LTA − TNFRSF1B
LTA − TNFRSF14
LTA − (LTB+LTBR)
KITL − KIT
INHBA − (ACVR1B+ACVR2B)
INHBA − (ACVR1B+ACVR2A)
IL7 − (IL7R+IL2RG)
IL6 − (IL6R+IL6ST)
IL4 − IL4R
IL4 − (IL4R+IL2RG)
IL4 − (IL4R+IL13RA2)
IL4 − (IL4R+IL13RA1)
IL34 − CSF1R
IL3 − (IL3RA+CSF2RB)
IL2 − (IL2RB+IL2RG)
IL1B − IL1R2
IL1B − (IL1R1+IL1RAP)
IL1A − IL1R2
IL1A − (IL1R1+IL1RAP)
IL15 − (IL15RA+IL2RB)
IL13 − IL13RA2
IL13 − IL13RA1
IL13 − (IL4R+IL13RA2)
IL13 − (IL4R+IL13RA1)
HGF − MET
HBEGF − ERBB4
HBEGF − EGFR
HBEGF − (ERBB2+ERBB4)
HBEGF − (EGFR+ERBB2)
GH1 − GHR
GH − PRLR
GH − GHR
GDNF − GFRA1
GAS6 − AXL
FLT3L − FLT3
FGF8 − FGFR1
FGF5 − FGFR1
FGF4 − FGFR1
EPO − EPOR
EGF − EGFR
EGF − (EGFR+ERBB2)
CXCL8 − CXCR2
CXCL3 − CXCR2
CXCL2 − CXCR2
CXCL12 − CXCR4
CXCL1 − CXCR2
CSF3 − CSF3R
CSF2 − (CSF2RA+CSF2RB)
CSF1 − CSF1R
CNTF − (CNTFR+LIFR)
CD40LG − (ITGAM+ITGB2)
CCL5 − CCR5
CCL5 − CCR3
CCL5 − CCR1
CCL4 − CCR5
CCL3 − CCR5
CCL3 − CCR1
CCL27 − CCR3
CCL27 − CCR2
CCL2 − CCR2
CCL14 − CCR1
CCL11 − CCR3
C3 − (ITGAX+ITGB2)
C3 − (ITGAM+ITGB2)
BTLA − TNFRSF14
BMP4 − (BMPR1B+ACVR2B)
BMP2 − (BMPR1B+ACVR2B)
BDNF − NTRK2
AREG − EGFR
AREG − (EGFR+ERBB2)
ANGPT2 − (ITGA5+ITGB1)
Endothelial cell −> Myeloid cell
Fibroblast −> Myeloid cell
Lymphocyte −> Myeloid cell
Keratinocyte 1 −> Myeloid cell
Smooth muscle −> Myeloid cell
Myeloid cell −> Myeloid cell
Keratinocyte 2 −> Myeloid cell
Melanocyte −> Myeloid cell
Commun. Prob.
max
min
p−value
0.01 < p < 0.05
p < 0.01

Fig S8

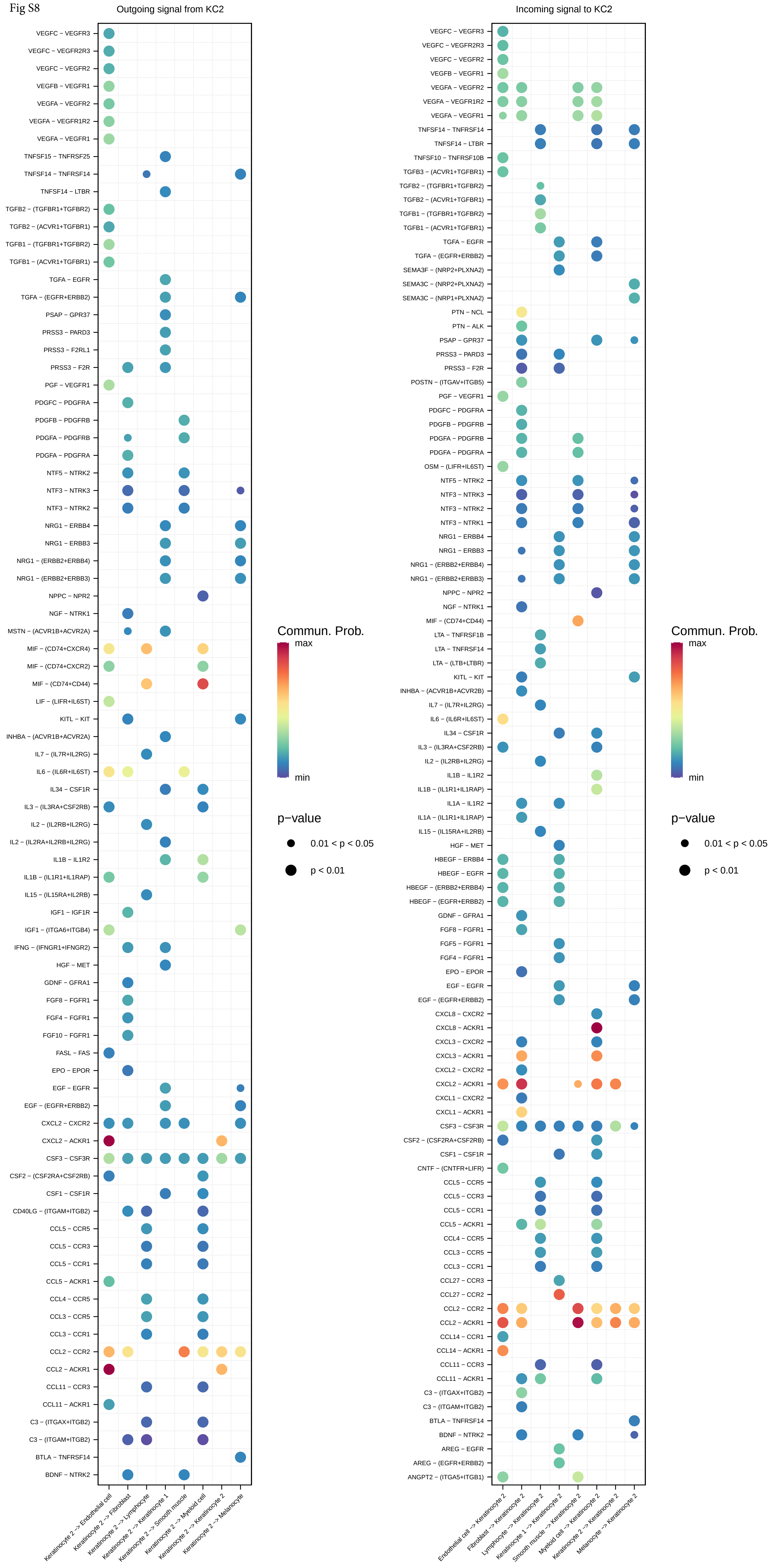

Outgoing signal from KC2
VEGFC − VEGFR3
VEGFC − VEGFR2R3
VEGFC − VEGFR2
VEGFB − VEGFR1
VEGFA − VEGFR2
VEGFA − VEGFR1R2
VEGFA − VEGFR1
TNFSF15 − TNFRSF25
TNFSF14 − TNFRSF14
TNFSF14 − LTBR
TGFB2 − (TGFBR1+TGFBR2)
TGFB2 − (ACVR1+TGFBR1)
TGFB1 − (TGFBR1+TGFBR2)
TGFB1 − (ACVR1+TGFBR1)
TGFA − EGFR
TGFA − (EGFR+ERBB2)
PSAP − GPR37
PRSS3 − PARD3
PRSS3 − F2RL1
PRSS3 − F2R
PGF − VEGFR1
PDGFC − PDGFRA
PDGFB − PDGFRB
PDGFA − PDGFRB
PDGFA − PDGFRA
NTF5 − NTRK2
NTF3 − NTRK3
NTF3 − NTRK2
NRG1 − ERBB4
NRG1 − ERBB3
NRG1 − (ERBB2+ERBB4)
NRG1 − (ERBB2+ERBB3)
NPPC − NPR2
NGF − NTRK1
MSTN − (ACVR1B+ACVR2A)
MIF − (CD74+CXCR4)
MIF − (CD74+CXCR2)
MIF − (CD74+CD44)
LIF − (LIFR+IL6ST)
KITL − KIT
INHBA − (ACVR1B+ACVR2A)
IL7 − (IL7R+IL2RG)
IL6 − (IL6R+IL6ST)
IL34 − CSF1R
IL3 − (IL3RA+CSF2RB)
IL2 − (IL2RB+IL2RG)
IL2 − (IL2RA+IL2RB+IL2RG)
IL1B − IL1R2
IL1B − (IL1R1+IL1RAP)
IL15 − (IL15RA+IL2RB)
IGF1 − IGF1R
IGF1 − (ITGA6+ITGB4)
IFNG − (IFNGR1+IFNGR2)
HGF − MET
GDNF − GFRA1
FGF8 − FGFR1
FGF4 − FGFR1
FGF10 − FGFR1
FASL − FAS
EPO − EPOR
EGF − EGFR
EGF − (EGFR+ERBB2)
CXCL2 − CXCR2
CXCL2 − ACKR1
CSF3 − CSF3R
CSF2 − (CSF2RA+CSF2RB)
CSF1 − CSF1R
CD40LG − (ITGAM+ITGB2)
CCL5 − CCR5
CCL5 − CCR3
CCL5 − CCR1
CCL5 − ACKR1
CCL4 − CCR5
CCL3 − CCR5
CCL3 − CCR1
CCL2 − CCR2
CCL2 − ACKR1
CCL11 − CCR3
CCL11 − ACKR1
C3 − (ITGAX+ITGB2)
C3 − (ITGAM+ITGB2)
BTLA − TNFRSF14
BDNF − NTRK2
Keratinocyte 2 −> Endothelial cell
Keratinocyte 2 −> Fibroblast
Keratinocyte 2 −> Lymphocyte
Keratinocyte 2 −> Keratinocyte 1
Keratinocyte 2 −> Smooth muscle
Keratinocyte 2 −> Myeloid cell
Keratinocyte 2 −> Keratinocyte 2
Keratinocyte 2 −> Melanocyte
Commun. Prob.
max
min
p−value
0.01 < p < 0.05
p < 0.01
Incoming signal to KC2
VEGFC − VEGFR3
VEGFC − VEGFR2R3
VEGFC − VEGFR2
VEGFB − VEGFR1
VEGFA − VEGFR2
VEGFA − VEGFR1R2
VEGFA − VEGFR1
TNFSF14 − TNFRSF14
TNFSF14 − LTBR
TNFSF10 − TNFRSF10B
TGFB3 − (ACVR1+TGFBR1)
TGFB2 − (TGFBR1+TGFBR2)
TGFB2 − (ACVR1+TGFBR1)
TGFB1 − (TGFBR1+TGFBR2)
TGFB1 − (ACVR1+TGFBR1)
TGFA − EGFR
TGFA − (EGFR+ERBB2)
SEMA3F − (NRP2+PLXNA2)
SEMA3C − (NRP2+PLXNA2)
SEMA3C − (NRP1+PLXNA2)
PTN − NCL
PTN − ALK
PSAP − GPR37
PRSS3 − PARD3
PRSS3 − F2R
POSTN − (ITGAV+ITGB5)
PGF − VEGFR1
PDGFC − PDGFRA
PDGFB − PDGFRB
PDGFA − PDGFRB
PDGFA − PDGFRA
OSM − (LIFR+IL6ST)
NTF5 − NTRK2
NTF3 − NTRK3
NTF3 − NTRK2
NTF3 − NTRK1
NRG1 − ERBB4
NRG1 − ERBB3
NRG1 − (ERBB2+ERBB4)
NRG1 − (ERBB2+ERBB3)
NPPC − NPR2
NGF − NTRK1
MIF − (CD74+CD44)
LTA − TNFRSF1B
LTA − TNFRSF14
LTA − (LTB+LTBR)
KITL − KIT
INHBA − (ACVR1B+ACVR2B)
IL7 − (IL7R+IL2RG)
IL6 − (IL6R+IL6ST)
IL34 − CSF1R
IL3 − (IL3RA+CSF2RB)
IL2 − (IL2RB+IL2RG)
IL1B − IL1R2
IL1B − (IL1R1+IL1RAP)
IL1A − IL1R2
IL1A − (IL1R1+IL1RAP)
IL15 − (IL15RA+IL2RB)
HGF − MET
HBEGF − ERBB4
HBEGF − EGFR
HBEGF − (ERBB2+ERBB4)
HBEGF − (EGFR+ERBB2)
GDNF − GFRA1
FGF8 − FGFR1
FGF5 − FGFR1
FGF4 − FGFR1
EPO − EPOR
EGF − EGFR
EGF − (EGFR+ERBB2)
CXCL8 − CXCR2
CXCL8 − ACKR1
CXCL3 − CXCR2
CXCL3 − ACKR1
CXCL2 − CXCR2
CXCL2 − ACKR1
CXCL1 − CXCR2
CXCL1 − ACKR1
CSF3 − CSF3R
CSF2 − (CSF2RA+CSF2RB)
CSF1 − CSF1R
CNTF − (CNTFR+LIFR)
CCL5 − CCR5
CCL5 − CCR3
CCL5 − CCR1
CCL5 − ACKR1
CCL4 − CCR5
CCL3 − CCR5
CCL3 − CCR1
CCL27 − CCR3
CCL27 − CCR2
CCL2 − CCR2
CCL2 − ACKR1
CCL14 − CCR1
CCL14 − ACKR1
CCL11 − CCR3
CCL11 − ACKR1
C3 − (ITGAX+ITGB2)
C3 − (ITGAM+ITGB2)
BTLA − TNFRSF14
BDNF − NTRK2
AREG − EGFR
AREG − (EGFR+ERBB2)
ANGPT2 − (ITGA5+ITGB1)
Endothelial cell −> Keratinocyte 2
Fibroblast −> Keratinocyte 2
Lymphocyte −> Keratinocyte 2
Keratinocyte 1 −> Keratinocyte 2
Smooth muscle −> Keratinocyte 2
Myeloid cell −> Keratinocyte 2
Keratinocyte 2 −> Keratinocyte 2
Melanocyte −> Keratinocyte 2
Commun. Prob.
max
min
p−value
0.01 < p < 0.05
p < 0.01

Fig S9

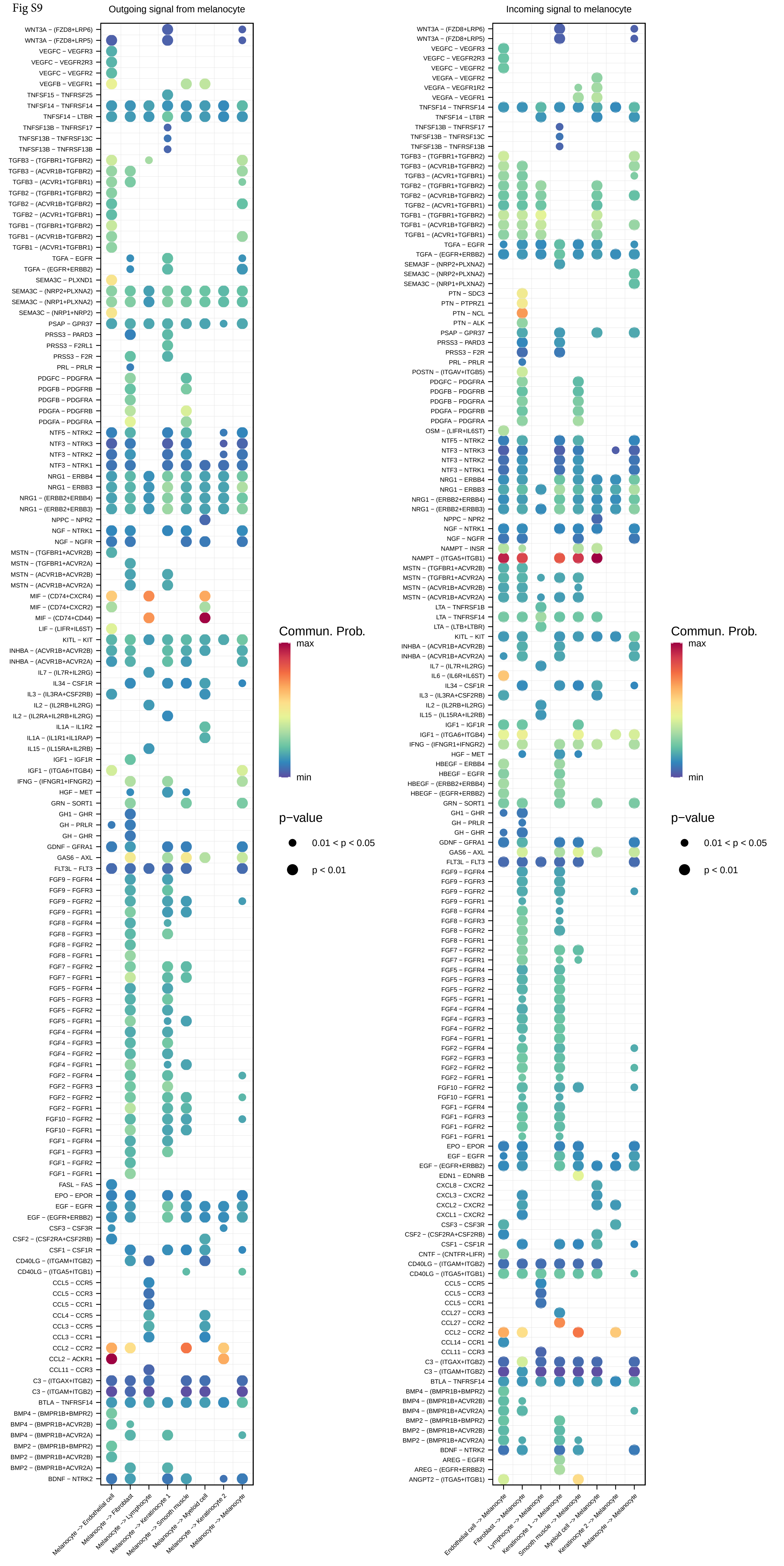

Outgoing signal from melanocyte
Incoming signal to melanocyte
Commun. Prob.
max
min
p−value
0.01 < p < 0.05
p < 0.01
Commun. Prob.
max
min
p−value
0.01 < p < 0.05
p < 0.01
