# Supplementary material for: Analysis of intracellular communication reveals consistent gene changes associated with early-stage acne skin
Source: Cell Commun Signal. 2024 Aug 14;22:400. doi: 10.1186/s12964-024-01725-4 (PMC11325718; doi:10.1186/s12964-024-01725-4)
Supplement: Supplementary file 15 — Supplementary Material 15 [file 12964_2024_1725_MOESM15_ESM.pdf]

Figure S1. Identification of cell markers and analysis of inferred signaling networks in Normal Skin Samples. A) Heatmap showing the cell markers for each cell type. **B**) Violin plot showing the cell markers for KC1 and KC2, comparatively. **C**) Circle plots illustrating the inferred signaling networks sent from each cell type to others. The edge width is proportional to the indicated number of interaction between cell types.

Figure S2. Comprehensive analysis of significant ligand-receptor pairs for endothelial cells from normal skin samples. Detailed representations of significant ligand-receptor pairs contributing to the outgoing (left side) and incoming (right side) signaling in endothelial cells. Dot colors and sizes indicate the communication probability and p-values, respectively, with p-values calculated using a one-sided permutation test.

Figure S3. Comprehensive analysis of significant ligand-receptor pairs for fibroblasts from normal skin samples. Detailed representations of significant ligand-receptor pairs contributing to the outgoing (left side) and incoming (right side) signaling in fibroblasts. Dot colors and sizes indicate the communication probability and p-values, respectively, with p-values calculated using a one-sided permutation test.

Figure S4. Comprehensive analysis of significant ligand-receptor pairs for lymphocytes from normal skin samples. Detailed representations of significant ligand-receptor pairs contributing to the outgoing (left side) and incoming (right side) signaling in lymphocytes. Dot colors and sizes indicate the communication probability and p-values, respectively, with p-values calculated using a one-sided permutation test.

Figure S5. Comprehensive analysis of significant ligand-receptor pairs for KC1 from normal skin samples. Detailed representations of significant ligand-receptor pairs contributing to the outgoing (left side) and incoming (right side) signaling in KC1. Dot colors and sizes indicate the communication probability and p-values, respectively, with p-values calculated using a one-sided permutation test.

Figure S6. Comprehensive analysis of significant ligand-receptor pairs for smooth muscle cells from normal skin samples. Detailed representations of significant ligand-receptor pairs contributing to the outgoing (left side) and incoming (right side) signaling in smooth muscle cells. Dot colors and sizes indicate the communication probability and p-values, respectively, with p-values calculated using a one-sided permutation test.

Figure S7. Comprehensive analysis of significant ligand-receptor pairs for myeloid cells from normal skin samples. Detailed representations of significant ligand-receptor pairs contributing to the outgoing (left side) and incoming (right side) signaling in myeloid cells. Dot colors and sizes indicate the communication probability and p-values, respectively, with p-values calculated using a one-sided permutation test.

Figure S8. Comprehensive analysis of significant ligand-receptor pairs for KC2 from normal skin samples Detailed representations of significant ligand-receptor pairs contributing to the outgoing (left side) and incoming (right side) signaling in KC2. Dot colors and sizes indicate the communication probability and p-values, respectively, with p-values calculated using a one-sided permutation test.

Figure S9. Comprehensive analysis of significant ligand-receptor pairs for melanocytes from normal skin samples Detailed representations of significant ligand-receptor pairs contributing to the outgoing (left side) and incoming (right side) signaling in melanocytes. Dot colors and sizes indicate the communication probability and p-values, respectively, with p-values calculated using a one-sided permutation test.

Figure S10. The top 10 L-R pairs in each cell type and specific ligands and receptors from skin datasets. **A**) The top 10 L-R pairs from each cell type based on their relative contribution under outgoing patterns. **B**) Dot plots showing specific expressed ligands or receptors genes derived from top 10 ligand-receptor pairs and top 3 outgoing and incoming signals for each cell type from nonlesional dataset of acne. **C**) Dot plots showing the expression pattern specific ligands and receptors for each cell type from the leprosy reversal reactions dataset [1]. The color scale represents the scaled expression average of each gene, while the dot size represents the percentage of cells expressing each gene.

Figure S11. Expression patterns of specific ligands and receptors relevant to endothelial cells and from Human Protein Atlas site. A-B) We began by analyzing ligand-receptor (L-R) expression based on a dataset by Tran et al. [2], and further validated the genes we identified using two additional datasets, the Leprosy and Human Protein atlas site [1, 3]. Panel A illustrates the expression levels of three specific ligands (CCL14, FLT1, CSF3), and Panel B shows the expression of three receptors (ACKR1, LIFR, TGFBR2), with values in normalized transcripts per million (nTPM).

Figure S12. The expression pattern of specific ligands for fibroblasts from Human Protein Atlas site [3]. The expression level of specific ligands (*CXCL12, PTN, C3, FGF7*) for fibroblasts are shown, with values in nTPM.

Figure S13. The expression pattern of specific receptors for fibroblasts from the Human Protein Atlas site [3]. The expression level of our specific receptors (*PDGFRA, FGFR1, SDC2, ACVR1*) for fibroblasts are shown, with values in nTPM.

Figure S14. Expression pattern of specific ligands and receptors for lymphocytes from Human Protein Atlas site [3]. A-C) Representation of the expression levels of two specific ligands (*TGFB1, CCL5*) (A) and four specific receptors (*CXCR4, IL7R, LTB, IL2RG*) (B and C) for lymphocytes, with values in in nTPM.

Figure S15. Expression pattern of specific ligands for myeloid cells from Human Protein Atlas site [3]. The expression level of specific ligands (*NAMPT, CXCL8, IL1B, VEGFA, CXCL3*) for myeloid cells, with values in nTPM.

Figure S16. Expression pattern of specific receptors for myeloid cells from Human Protein Atlas site [3]. The expression level of specific receptors (*CD74, CD44, IL1R2, ITGAX*) for myeloid cells, with values in nTPM.

Figure S17. Expression pattern of specific ligands and receptors for KCs and smooth muscle cells from Human Protein Atlas site [3]. A-C) The expression level of specific ligand (*AREG*) and receptors (*EGFR* and *ERBB2*) for KCs (A-B), and ligand (*PDGFA*) for smooth muscle cells (C), with values in nTPM.

Figure S18. Analysis of interaction differences between nonlesional and lesional samples. A) Comparative analysis of the total number (left) and weights (right) of potential interactions across all cell types between nonlesional and lesional samples. **B)** Circular plots depicting changes in the total number of interactions (left) and their weights (right) across individual cell clusters between nonlesional and lesional samples.

Figure S19. Differential gene expression in nonlesional and lesional samples within each patient. Violin plots illustrating the expression of 26 genes that exhibit significant differences between nonlesional and lesional samples within individual patients. Wilcoxon test was used for statistical analysis, with significance levels marked as, *$P < 0.05$, **$P < 0.01$, ***$P < 0.001$, ****$P < 0.0001$.

Figure S20. Subsets and comparative UMAP Analysis of Myeloid Cells, Keratinocytes, and Lymphocytes. **A, C, and E**) UMAP visualizations displaying subsets of myeloid cells (A), keratinocytes (C), and lymphocytes (E). **B, D, and F**) UMAP plots for myeloid cells (B), keratinocytes (D), and lymphocytes (F) from nonlesional and lesional samples, color-coded by lesion type; nonlesional cells are indicated in blue and lesional cells in red.

Figure S21. Colocalization analysis and gene expression in HaCaT cell line. A-B) Spatial feature plots showing overlay of *GRN* in red with *IL-1β* (A) or *TNF-α* (B) in green, with 2-μm intervals between grids; the boxed region highlights a magnified view of the spatial plot. **C)** qRT-PCR analysis of *IL-13RA1* and *IL-4R* expression in HaCaT cells treated with various concentrations of IL-4 and PBS over 24 hours, with significance indicated as ***P < 0.001 (Student's t-test), and ns (not significant) for n = 3 donors. **D)** qRT-PCR analysis of *KRT6A* expression in HaCaT cells treated with 20 ng/ml of recombinant IL-13 protein and PBS for 24 hours, noted as ns (not significant), for n = 3 donors.

REFERENCES

1. Ma, F. *et al.* The cellular architecture of the antimicrobial response network in human leprosy granulomas. *Nat Immunol* **22**, 839-850 (2021).
2. Do, T.H. *et al.* TREM2 macrophages induced by human lipids drive inflammation in acne lesions. *Sci Immunol* **7**, eabo2787 (2022).
3. Karlsson, M. *et al.* A single-cell type transcriptomics map of human tissues. *Sci Adv* **7** (2021).
